# Improving access to fertility treatment in Asia Pacific: consensus from an expert forum

**DOI:** 10.3389/frph.2026.1738726

**Published:** 2026-03-20

**Authors:** Marinella Agnes G. Abat, Hannah Armstrong, Mei-Jou Chen, Human Fatemi, Ho Sy Hung, Koji Nakagawa, Navdeep Singh Pannu, Angelina Petrova, Michele Pistollato, Kamthorn Pruksananonda, Jung Ryeol Lee, Tze Tein Yong

**Affiliations:** 1 Center for Advanced Reproductive Medicine and Infertility (CARMI) St. Luke’s Medical Center Global City, Taguig City, Philippines; 2 GenPrime Manila, Manila, Philippines; 3 Life Sciences Policy, Charles River Associates, London, United Kingdom; 4 Department of Obstetrics and Gynecology, National Taiwan University Hospital, Taipei City, Taiwan; 5 College of Medicine, National Taiwan University, Taipei, Taiwan; 6 ART Fertility Clinics, Abu Dhabi, United Arab Emirates; 7 Obstetrics and Gynecology Department, Hanoi Medical University, Hanoi, Vietnam; 8 Sugiyama Clinic, Shinjuku Reproductive Medical Center, Tokyo, Japan; 9 Alpha IVF & Women’s Specialist Centre, Petaling Jaya, Malaysia; 10 Chulalongkorn University, Bangkok, Thailand; 11 Department of Obstetrics and Gynaecology, Seoul National University Bundang Hospital, Seoul, Republic of Korea; 12 Seoul National University College of Medicine, Seoul, Republic of Korea; 13 Department of Obstetrics and Gynaecology, Singapore General Hospital, Singapore

**Keywords:** Asia Pacific, assisted reproduction technology, egg freezing, fertility policy, fertility treatment, infertility, policy recommendations

## Abstract

**Background:**

Declining fertility rates are a globally documented problem, affecting even regions that have historically high fertility rates. In the Asia Pacific (APAC) region, most countries and territories are seeing birth rates drop far below the replacement rate of 2.1 children per woman. Policymakers in these countries have switched from a historic focus on population control policies to investing in pronatalist policies that aim to boost the national fertility rate by tackling the socioeconomic drivers of falling fertility. In this paper, we examine policies affecting the often-overlooked medical aspect of this challenge: infertility.

**Objective:**

To review challenges in fertility policy and propose solutions to improve equitable access to assisted reproductive technologies (ART) and associated services.

**Methods:**

A review of literature was conducted on fertility policy challenges and developments in the APAC region, covering fertility recognition and awareness; egg freezing; and access to ART treatment, psychosocial care, and supplementary care. Experts from nine different countries and territories were invited to validate secondary research findings and provide their perspectives on policy implications. The experts participated in individual 60-minute interviews, then a half-day Policy Forum discussion was held at the Asia-Pacific Assisted Conception Congress 2024, held in Kuala Lumpur, Malaysia.

**Results:**

Whilst several countries in the region have long recognised falling total fertility rates (TFR) as a cause for concern, many have been slow to recognise infertility as a disease, resulting in limited policy development to support patients experiencing infertility. Commonly this presents as lack of funding allocated towards fertility services, limited or no reimbursement of fertility treatment or egg freezing, and poor societal awareness of infertility and hence delays in seeking care. Various policy changes can be adopted to improve care for patients across the APAC region.

**Conclusion:**

Our review identified multiple gaps in policy development for supporting individuals experiencing infertility. Looking to countries in which the TFR dropped below replacement level many years ago, the importance of timely policy intervention before negative demographic consequences occur is critical.

## 1 Introduction

The challenge of falling fertility in the Asia Pacific (APAC) region is well documented. Most countries and territories in the region have total fertility rates (TFRs) near or below the replacement level of 2.1, meaning populations are expected to begin declining (1). Declining TFR carries significant socioeconomic risks as it contributes to an ageing population and population decline. For instance, in Japan, the TFR in the 1980s was 1.69 (already below replacement level), has only continued to decrease (2). As a result, the size of the working-age population in Japan is projected to decrease from 81 million in 2010 to 44 million in 2060 (3). This decline has had a negative impact on the productivity of the Japanese economy (4).

To boost fertility rates, many governments have switched their public policy focus from family planning objectives aimed at curbing population growth to pronatalist policies aimed at raising birth rates. Ultimately, much of the observed fall in fertility rates is attributable to people choosing to have fewer children for various social, economic, and societal reasons. Combatting a population-level decline in the desire to have children is, therefore, extremely challenging. There is much work ongoing to assess and implement policies that support more “family-friendly” societies (5). At the same time, there are many people who do want to have children but are unable to do so: those experiencing infertility. However, there are no existing studies that provide a detailed review of infertility policies and treatment access across the APAC region.

**Figure 1:**
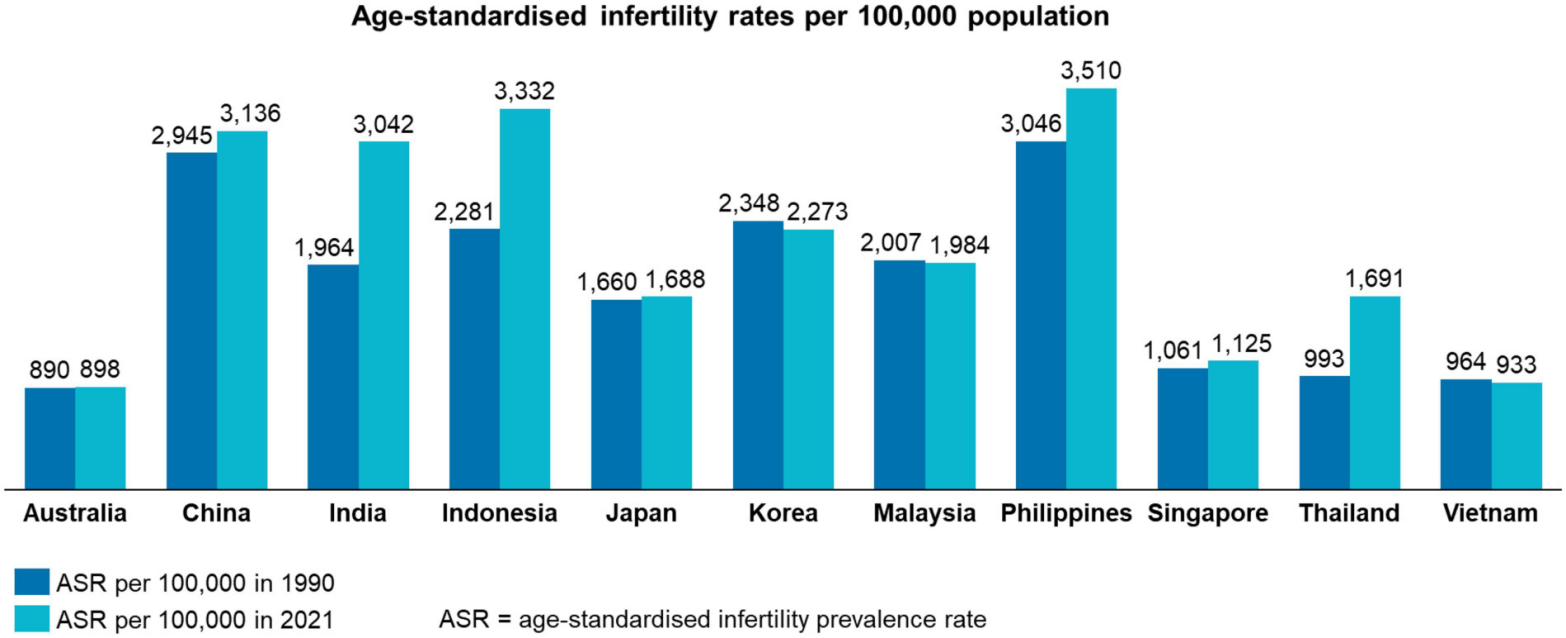
Infertility rates across the Asia Pacific region, 1990 and 2021. Luoet al. (7).

Globally, an estimated one out of every six people is affected by infertility (6). Rates of infertility vary between countries and territories across the APAC region (Figure 1). For example, recent analysis has found that the Philippines have the highest infertility rate in the region (7). For all women, infertility correlates closely with increasing age (8). The average childbearing age varies significantly across the APAC region. For example, women give birth to their first child on average at 21 years old in India, and at over 32 years old in Korea (9). Women in the region, therefore, experience the burden of age-related infertility to different extents. Other drivers of infertility include reproductive disorders such as endometriosis, uterine fibroids, and polycystic ovarian syndrome (PCOS). The incidence of PCOS varies significantly across the APAC region—for example, from 33 per 100,000 population in Korea to 97 per 100,000 in Taiwan—and hence, its level of influence on infertility rates also varies across the region (10).

The inability to achieve a pregnancy can have a substantial psychological impact on those experiencing infertility, significantly affecting their quality of life. In a study in Taiwan, 40% of women seeking treatment for infertility were diagnosed with a psychiatric disorder (most commonly anxiety and/or depression) (11). A longitudinal study in Australia found similar results: half of women experiencing infertility reported significant psychological distress (12). The economic and cultural context within Asian countries and territories can exacerbate these effects. For instance, in Chinese culture, many married couples perceive having children as an honour and duty to continue the family lineage. Chinese couples experiencing infertility, therefore, bear a significant emotional burden related to both their individual psychological stress and due to wider social pressure (13). Similar evidence has been found in Vietnam, where both the husband and wife experience equivalent psychological distress, irrespective of whether the cause of the infertility is male or female, because of their shared pressure to start a family (14).

In addition to the direct impact on the quality of life of those experiencing infertility, another spillover effect of this is reduced participation in the workforce. For instance, a Japanese study assessing the impact of fertility treatment on working women found that women often experienced harassment in the workplace and were not provided with the necessary support, resulting in one-sixth of women resigning after starting infertility treatment (15). The socioeconomic burden of infertility is therefore felt at both an individual and a societal level.

**Figure 2:**
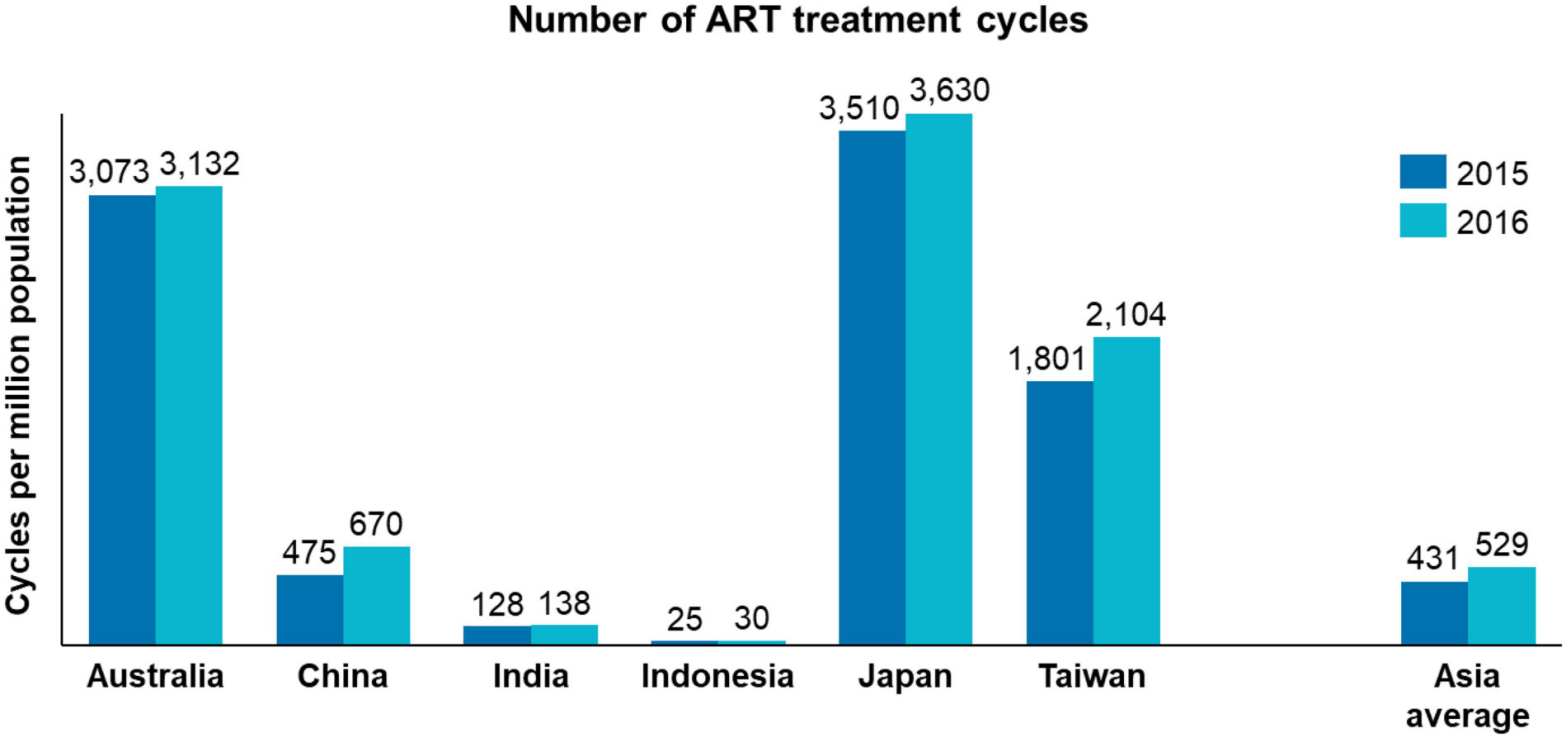
Annual number of ART treatment cycles conducted across countries, ICMART, 2024. Kupka et al. ART, assisted reproductive technology. (17).

There are various treatment options for infertility. In this paper, when we examine access to treatment, we primarily refer to assisted reproductive technology (ART); we separately consider egg freezing. ART is a term that encompasses all fertility treatments in which eggs, sperm or embryos are handled *in vitro* for the purpose of establishing a pregnancy (16). Global data on ART use are collected and analysed by the International Committee for Monitoring Assisted Reproductive Technologies (ICMART). Limited data are available in the APAC region, but for the countries and territories where there are recent data, we can observe an apparent disparity (as shown in Figure 2). ART utilisation has been recognised as an indicator of access to infertility care that can inform and evaluate the success of fertility policy initiatives (18). The disparity observed in (7).

Figure 2 is therefore indicative of significant inequity of access to infertility care across the APAC region and suggests variable levels of policy development. One factor that has been found to influence ART utilisation is financial accessibility (18). It is unsurprising that in (7).

Figure 2 we observe that the larger APAC economies, in which there is generally some level of public reimbursement of ART, have the highest utilisation (19). Previous studies in low- and middle-income economies have found that ART typically receives no government funding or only partial subsidisation and, therefore, remains unaffordable for most patients. For instance, analysis has suggested that the medical cost for just one ART cycle in South-East Asian countries and territories is, on average, 327% higher than the average gross domestic product (GDP) per capita (20). In this research we investigate the factors directly affecting access to ART (such as public reimbursement) as well as other factors that indirectly affect access to ART and fertility care (such as recognition and awareness, availability of psychosocial support, and use of supplementary care).

## 2 Methods

This study employed a structured narrative review combined with a multi-stage expert insight gathering process to examine policy gaps and identify priority areas for improving equitable access to ART across the APAC region. The methodological design was chosen to synthesise heterogeneous evidence sources, peer-reviewed literature, policy documents, grey literature, and expert insights, and to generate contextually grounded policy recommendations.

### 2.1 Structured narrative review

A structured narrative review was conducted to consolidate evidence on fertility-related policies, barriers to treatment access, and emerging best practices across the APAC region. This approach was selected due to the diversity and limited standardisation among available sources, which precluded systematic meta-analysis yet required a transparent synthesis process. The review commenced with an exploratory scoping phase aimed at mapping the breadth of fertility-policy literature and identifying recurring policy themes relevant to ART access. This phase drew upon global reproductive-policy frameworks, most notably the International Federation of Fertility Societies (IFFS) Surveillance 2022 report, which provides an internationally recognised taxonomy for categorising reproductive-health policies (21). Insights from this scoping phase informed the development of the analytic framework used throughout the study. Additionally, a paper previously published by a subset of the coauthors, was used to inform the framework of the study (22); it also relied on the IFFS 2022 taxonomy.

Five thematic domains were identified, integrating patterns observed in the preliminary scoping review and policy categories highlighted by the IFFS Surveillance 2022 report. These domains were selected because they represent core structural determinants of access to infertility care and ART across diverse health-system contexts:

- **Recognition and awareness** of infertility as a disease, including public awareness and political prioritisation.
- **Egg freezing policies**, covering both medical and social oocyte cryopreservation.
- **Access to fertility treatment**, including geographic availability of ART centres and financial accessibility through reimbursement or subsidies.
- **Psychosocial support**, referring to psychological care embedded in the infertility and ART treatment pathway.
- **Supplementary care**, including access to preimplantation genetic testing (PGT-M and PGT-SR) and governance of non-validated ART add-ons.

The five thematic domains were selected based on their recurrence in the scoping review, alignment with internationally recognised reproductive health policy taxonomies, and relevance as modifiable policy levers within infertility care systems. Collectively, they capture key structural determinants along the medical infertility care pathway, from societal and political recognition of infertility, through entry points into care, to the availability, quality, and governance of ART-related services. Recognition and awareness influence prioritisation and resource allocation; egg freezing policies reflect a rapidly evolving area of reproductive governance; access to fertility treatment encompasses geographic and financial barriers; psychosocial support addresses the substantial mental health burden associated with infertility; and supplementary care reflects regulatory approaches to advanced and adjunctive ART interventions. Together, these domains provide a focused framework for cross-jurisdictional comparison across diverse health system contexts in the APAC region. Broader socioeconomic or pronatalist themes, such as childcare provision, workplace protections, housing affordability, cultural norms, or individual fertility intentions, were excluded, as these factors primarily shape reproductive decision-making and fertility demand rather than directly regulating access to infertility diagnosis or ART services. Including such domains would have expanded the scope beyond health system governance. The analytic framework therefore reflects a purposeful methodological choice to concentrate on policies that directly enable or constrain access to medical infertility care, while acknowledging that these policies operate within a wider social and economic environment that warrants separate, complementary investigation.

The literature search covered the period 2014–2024 and was conducted using Google Scholar, complemented by targeted searches in local languages where feasible. Keyword combinations included “*assisted reproductive technology”, “fertility policy”, “egg freezing”, “fertility awareness”, “psychosocial care”, “Asia Pacific”, “challenges”*, and “*best practices*”. The search aimed to capture both academic and grey literature relevant to fertility-policy design and implementation. Through this structured process, 105 publications were selected for qualitative synthesis. These sources informed the assessment of policy development across the countries and territories included in the review: Australia, China, Hong Kong SAR (China), India, Indonesia, Japan, Malaysia, Philippines, Singapore, Korea, Taiwan, Thailand, and Vietnam. These jurisdictions were chosen to reflect a broad spectrum of TFRs, economic contexts, and approaches to fertility policy.

### 2.2 Expert insight gathering

To complement and validate the evidence from the literature review, a structured two-stage expert-engagement process was undertaken.

Nine experts from nine countries and territories were purposively selected based on clinical, academic, and policy expertise, ensuring representation across: high-volume ART centres; academic research institutions; public and private fertility-care settings; and jurisdictions at varying stages of policy development. Semi-structured, 60 min interviews explored national policy contexts, perceived barriers to ART access, gaps in implementation, and views on effective policy models (Table 1). The final stage was to convene an in-person Policy Roundtable, which was facilitated by Hannah Armstrong and Angelina Petrova on 27 September 2024 in Kuala Lumpur, Malaysia. During the Policy Roundtable, the experts reviewed evidence of the barriers to ART access, discussed lessons that can be drawn from the identified “best practice” policies, and co-developed implementable and actionable (national and regional) policy goals to support optimal patient access and care. Consensus points were documented and integrated into the final synthesis.

**Table 1:** List of experts who participated in the one-to-one interviews.

Name	Place of residence	Affiliation
Dr. Marinella Agnes G Abat	Philippines	Centre for Advanced Reproductive Medicine and Infertility (CARMI) St. Luke's Medical Centre Global City; GenPrime Manila
Prof. Dr. Mei-Jou Chen	Taiwan	Department of Obstetrics and Gynecology, National Taiwan University Hospital; College of Medicine, National Taiwan University, Taipei, Taiwan
Prof. Dr. Human Fatemi	United Arab Emirates	Group Medical Director of ART Fertility Clinics
Dr. Ho Sy Hung	Vietnam	Obstetrics and Gynecology Department, Hanoi Medical University
Prof. Dr. Jung Ryeol Lee	Korea	Department of Obstetrics and Gynaecology, Seoul National University Bundang Hospital, Seoul National University College of Medicine
Dr. Koji Nakagawa	Japan	Managing Director of Sugiyama Clinic Shinjuku, Tokyo, Japan
Prof. Dr. Kamthorn Pruksananonda	Thailand	Chulalongkorn University
Dr. Navdeep Singh Pannu	Malaysia	Alpha IVF & Women's Specialist Centre, Malaysia
A/Prof. Tze Tein Yong	Singapore	Department of Obstetrics and Gynaecology, Singapore General Hospital

To enable cross-country comparison, each jurisdiction was rated qualitatively (red–amber–green) across the five thematic domains. Scoring was based on evidence of political prioritisation, regulatory robustness, and existence of public funding mechanisms. These ratings were intended as directional indicators rather than quantitative metrics and were used to guide the tailoring of recommendations to different levels of policy maturity.

Although the study was funded by Organon International, all aspects of evidence synthesis, methodological design, analysis, and manuscript drafting were led independently by the authors. Expert opinions were incorporated through structured processes designed to minimise bias, and the sponsor did not influence analytic decisions.

## 3 Challenges in accessing egg freezing, fertility treatment and care

The findings of the cross-country policy review have been structured into five categories, the scope of which are specified below:

- **Recognition and awareness:** The extent to which policymakers recognise infertility as a disease and provide appropriate policy support and funding; existence of public awareness and educational campaigns on infertility
- **Egg freezing:** Policies impacting access to egg freezing, for both medical and personal reasons (whether this is permitted, and whether any public reimbursement or subsidisation is available)
- **Access to fertility treatment:** Policies impacting access to IVF and other forms of fertility treatment [availability of centres and healthcare professionals (HCPs), reimbursement and subsidisation policies]
- **Psychosocial support:** The availability and reimbursement of any psychosocial care for patients undergoing fertility treatment
- **Use of supplementary care:** Policies impacting availability of and access to preimplantation genetic testing (PGT) for patients undergoing ART treatment; regulations or information campaigns surrounding non-validated “add-ons” to ART.

### 3.1 Recognition and awareness

#### 3.1.1 Political recognition of infertility

Evidence suggests that APAC countries and territories have been relatively slow to recognise infertility as a disease, due to a combination of socioeconomic and cultural factors. There is significant variation in TFRs in the region, with a high of 2.18 in Indonesia and a low of 0.78 in Korea (23, 24); this may explain the different levels of prioritisation of infertility as a disease. In countries and territories with a low TFR and a history of prolonged decline, such as Korea and Japan, this has had the effect of boosting political recognition of infertility and catalysed targeted fertility policy development. However, in countries and territories where populations are stable or still growing, political recognition of infertility is much more limited. For instance, we note that in Vietnam, infertility is not recognised as disease by the Ministry of Health, as was emphasised during the Policy Roundtable. This is problematic, as irrespective of TFR (influenced by many socioeconomic factors), there will still be many couples and individuals experiencing infertility (a medical condition).

Under-recognition may be partially attributable to the historical context. Throughout much of the 20th century, many APAC countries and territories implemented proactive family planning policies aimed at reducing birth rates to curb rapid population growth. Countries such as China (25), India (26), and Indonesia pursued these policies because of concerns over resource scarcity, economic development, and environmental sustainability (27). Similar approaches were adopted elsewhere in Southeast Asia: Thailand and Malaysia introduced national family planning programmes in the 1960s and 1970s that prioritised voluntary contraception and public education to rapidly reduce fertility rates, while Vietnam implemented a more directive two-child policy in the late 1980s to constrain post-war population growth (28–30). In the Philippines, although policy implementation was slower due to religious and political resistance, the eventual passage of the Responsible Parenthood and Reproductive Health Act similarly centred population management through access to contraception and reproductive health services (31).

In this context, fertility was seen as something to be carefully managed, and reproductive health policies were geared primarily toward contraception. As a result of this historical context, infertility is often downgraded in political perceptions to a lifestyle matter that stems from personal actions and choices. This has created unique cultural dynamics with respect to fertility policies which continues to affect how new policies are drafted and implemented. As will be covered in the subsequent sections, cultural norms impact restrictions on egg freezing and PGT.

Where governments have recognised infertility as a disease, there has often been a lack of follow-through policy development to support access to treatment, or there has been insufficient funding allocated to support fertility policy priorities. For instance, the Malaysian government has recognised infertility as a medical condition, but the country has seen limited policy development to support patient access to treatment. While ART treatments such as IVF are available in Malaysia, they are largely confined to the private healthcare sector, making them unaffordable for many individuals and couples (31). ART treatment is only subsidised in a select number of public health centres, located urbanely, leaving a significant gap between recognition of infertility as a disease and equitable access to affordable treatment (21). ART reimbursement will be discussed further in later sections.

Although Korea currently has a robust policy framework to support fertility, there was a significant delay in the policy response when the TFR initially fell below the replacement rate of 2.1 in 1983 (32). Based on discussions with experts and findings from literature, it took over 20 years for policymakers to draft the first policy framework to address the declining TFR (32). Nonetheless, it is important to highlight that fertility challenges, driven by the falling TFR, are becoming to be more widely discussed.

#### 3.1.2 Public awareness

Evidence highlights a fundamental policy challenge: many individuals remain unaware that infertility is a medical condition and lack basic understanding of its common causes and available treatments (33). Infertility is frequently perceived as an abstract or exceptional issue rather than a foreseeable health risk, and its key drivers, such as age, lifestyle factors, and underlying medical conditions, are poorly understood by the general population. These knowledge gaps contribute to persistent misconceptions, including the widespread belief that infertility is primarily or exclusively a female issue, despite the well-established role of male infertility (33).

A growing body of literature suggests that these gaps in fertility awareness across the APAC are closely linked to limitations in the scope and timing of reproductive health education provided to individuals of childbearing age. Assessments by the United Nations Educational, Scientific and Cultural Organization (UNESCO) and the United Nations Population Fund (UNFPA) show that topics such as infertility are largely absent across much of East, Southeast, and South Asia, reflecting curricular caution (34).

The consequences of these educational gaps are evident in empirical studies from East Asia. Surveys conducted in Japan and Korea consistently find that adults, including those with tertiary education, overestimate the age at which natural fertility remains high and underestimate the speed of reproductive decline, particularly for women (35, 36). In Japan, women who reported having accurate fertility knowledge in early adulthood were significantly more likely to transition to parenthood at younger ages, indicating a direct association between timely fertility information and reproductive behaviour (37).

Deficits in fertility awareness also shape health-seeking behaviour and access to care. Public understanding of the drivers of infertility influences both whether and how quickly individuals seek formal diagnosis and medical support. For example, a study in India found that women with higher levels of reproductive health knowledge were more likely to recognise infertility as a biological and medical condition and, consequently, more likely to seek medical treatment (38). In contrast, multiple studies indicate that women in the APAC region tend to wait longer before seeking infertility treatment compared with women in other regions (39). In Taiwan, recent evidence shows that the average time to infertility diagnosis is 2.9 years (40). Given that infertility is an age-sensitive condition, such delays can substantially reduce the likelihood of successful treatment outcomes (41). Notably, research examining levels of infertility awareness remains sparse in several countries, including the Philippines, Indonesia, India, and Vietnam.

Inequalities in fertility awareness further compound these challenges. Studies consistently show that fertility awareness in the APAC region is higher in urban than in rural populations, reflecting greater exposure to education, healthcare services, and reproductive health information in urban settings. In rural communities, limited access to information means individuals are often less aware of fertility preservation options, including ART and egg freezing. For instance, a recent study in Indonesia found that rural respondents had a poorer understanding of the definition of infertility and were more likely to hold misconceptions about the condition (42). These disparities have tangible consequences: evidence suggests that individuals living in rural areas have a lower likelihood of achieving successful fertility treatment outcomes (43).

### 3.2 Egg freezing

According to the American Society for Reproductive Medicine (ASRM), elective egg freezing, also referred to as oocyte cryopreservation, is a reproductive technology that allows women to preserve their eggs for medical reasons—such as potential fertility loss due to upcoming treatment—and non-medical reasons, such as delay of childbearing (44). While this technology has gained global recognition, policies governing egg freezing remain in their early stages across the APAC region. Below we will cover both broad types of egg freezing: medical and social.

#### 3.2.1 Availability of medical egg freezing

Certain medical treatments, particularly oncological therapies, pose significant risks to fertility and represent an important policy and clinical context for fertility preservation. Chemotherapy and radiotherapy administered near reproductive organs can damage oocytes and sperm, while surgical interventions involving reproductive tissues may directly compromise fertility (45). In addition, hormonal therapies commonly used in cancer care can disrupt endocrine function and negatively affect reproductive capacity over the long term. As a result, cancer treatment is a primary medical indication for fertility preservation and a key motivation for egg freezing prior to the initiation of therapy (41).

Although egg freezing offers a viable option for preserving reproductive potential before fertility-damaging treatment, access to this intervention remains uneven across the APAC region, in part due to limited awareness among both patients and healthcare professionals (HCPs). Many oncologists and other HCPs treating conditions that may impair fertility do not consistently discuss fertility preservation options, including egg freezing, with their patients. Insights from experts participating in the Policy Roundtable suggest that this gap may arise from time constraints during oncology consultations, a necessary prioritisation of cancer treatment, or limited familiarity with referral pathways and the availability of fertility specialists. Consequently, patients may not be referred early enough to explore egg freezing prior to commencing cancer treatment. Empirical evidence from Malaysia supports this observation; a study conducted in 2021 found that awareness of medical egg freezing among HCPs was limited, as reflected by low referral rates to oncofertility specialists before cancer treatment (46).

From the patient perspective, insufficient awareness further constrains access to fertility preservation. During the Policy Roundtable, experts highlighted that cancer patients frequently lack timely information about medical egg freezing as a fertility preservation option. This observation is supported by the literature: a study from Hong Kong identified a substantial unmet need for timely and comprehensive information on egg freezing among cancer patients (47). Similar findings were reported in in two studies from China (48, 49). Without adequate counselling at diagnosis or treatment planning stages, patients may lose the opportunity to preserve fertility before irreversible treatment effects occur.

Financial barriers compound these informational and referral challenges. Despite the clinical importance of medical egg freezing for preventing treatment-induced infertility, the procedure is often only partially reimbursed or not reimbursed at all by public healthcare systems in the APAC region. As shown in Table 2, less than half of APAC countries and territories in scope provide full or partial public reimbursement for medical egg freezing. For example, in Singapore, medical egg freezing is permitted and partially subsidised, even though ART treatments receive reimbursement through the public system (59, 63). Evidence from Hong Kong further demonstrates that lack of full reimbursement has a significant negative impact on access: financial constraints were identified as the most important reason patients did not pursue egg freezing prior to cancer treatment (47).

**Table 2:** Public funding of medical egg freezing across APAC.

Country/territory	Availability of public funding for medical egg freezing^a^
Australia (50)	Available through rebates
China (51)	Not available
Hong Kong (52)	Available through partial subsidisation
India (53)	Not available
Indonesia (42)	Not available
Japan (54)^b^	Available in certain prefectures and cities through subsidies
Korea (55, 56)^b^	Available nationally and locally through partial subsidisation
Malaysia (57)^b^	Not available
Philippines (58)^b^	Not available
Singapore (59)^b^	Available through partial subsidies
Taiwan (60)^b^	Available through partial subsidies
Thailand (61)^b^	Not available
Vietnam (62)^b^	Not available

^a^ Describes the public funding mechanism through which medically indicated egg freezing is supported by the government.

^b^ Confirmed with experts during Policy Roundtable.

Overall, these gaps in awareness, referral practices, and reimbursement limit patient access to medical egg freezing and reduce the likelihood that fertility preservation can be pursued in a timely manner before the initiation of fertility-damaging treatment.

#### 3.2.2 Availability of social egg freezing

Social egg freezing refers to the cryopreservation of oocytes undertaken to postpone childbearing for non-medical reasons (44). Women may pursue social egg freezing for a range of pragmatic reasons, including delays associated with financial instability or the absence of a suitable partner. Across many countries and territories in the APAC region, however, access to social egg freezing is constrained by regulatory and legal restrictions. In China, as presented in Table 3, the procedure is restricted to married women, reflecting policies strongly informed by cultural norms and traditional conceptions of family structure in which childbearing is expected to occur within heterosexual marriage. For example, policymakers in Singapore have expressed concern about the potential implications of social egg freezing for traditional family values (75). These regulatory approaches illustrate the substantial influence of cultural context on ART policy development and the resulting limitations on access to social egg freezing. It is important to note, that in most APAC countries and territories in scope (excluding Australia, the Philippines, India, and Vietnam), while single women increasingly may have access to egg freezing itself, the use of thawed eggs to conceive is either formally or practically restricted to married heterosexual couples (5).

**Table 3:** Availability of social egg freezing and existence of public funding mechanisms.

Country/territory	Availability of social egg freezing and/or existence of public funding mechanisms^a^
Australia (50)	Available; no public funding mechanisms
China (64, 65)	Available; only permitted for married women; no public funding mechanisms
Hong Kong (66)	Available; no public funding mechanisms
India (67)	Available; no public funding mechanisms
Indonesia (42)	Available; no public funding mechanisms
Japan (68)^b^	Available; subsidies are offered in certain prefectures and cities
Korea (69)^b^	Available; subsidies are offered to women aged 20 to 49 on a local basis (not nationally); requirements like AMH level or income limits apply
Malaysia (57, 70)^b^	Available; no public funding mechanisms
Philippines (71)^b^	Available; no public funding mechanisms
Singapore (72)^b^	Available for women at least 21 years of age but below 38 years of age; no public funding mechanisms
Taiwan (60)^b^	Available; subsidies are provided for women at least 25 years of age but below 40 years of age
Thailand (73)^b^	Available; no public funding mechanisms
Vietnam (74)^b^	Available; no public funding mechanisms

^a^ Describes the public funding mechanism through which social egg freezing is supported by the government.

^b^ Confirmed with experts during Policy Roundtable.

Such restrictions have uneven effects on different population groups. Women with sufficient financial resources may be able to circumvent national regulations by travelling to countries or territories where social egg freezing is permitted. In contrast, women from lower-income backgrounds or those living in rural areas often face significantly fewer options. In this way, restrictive policies reinforce societal norms that prioritise marriage as the sole acceptable context for childbearing and egg freezing, while limiting women's ability to exercise autonomous reproductive decision-making.

Beyond legal permissibility, access to social egg freezing is further shaped by the availability of public funding mechanisms, which varies considerably across the APAC region. As shown in Table 3, while social egg freezing is legally available in all jurisdictions, public subsidies are provided in only a small number of countries and territories, most notably Japan, Korea, and Taiwan. In Japan, funding is limited to selected prefectures and cities, whereas Korea and Taiwan offer more structured, though still age-restricted, support. By contrast, in Australia, Hong Kong, Singapore, and much of Southeast Asia, social egg freezing remains entirely self-funded.

The absence of public funding has direct implications for uptake and timing. Evidence from Australia indicates that financial barriers are among the key reasons women do not pursue social egg freezing (76). Where cost leads to delayed decision-making, women may freeze their eggs at older ages, when egg quality is already declining. Oocytes retrieved later in life are less likely to result in successful conception (77), thereby reducing the effectiveness of subsequent ART and decreasing the probability of successful fertilisation and pregnancy.

In combination, regulatory restrictions and the lack of public funding limit access to social egg freezing and delay the timing at which women are able to engage with fertility preservation services.

### 3.3 Access to fertility treatment

Access to fertility treatment (as measured by number of ART cycles) is highly variable across the APAC region. In the countries and territories where data are available, access to ART ranges from 3,630 annual cycles per million population in Japan to only 30 cycles per million population in Indonesia (17); this is more than a one-hundred-fold difference across the region. We explored two types of policies affecting patient access to treatment: availability of fertility treatment centres providing standardised treatment, and reimbursement and subsidisation of ART.

#### 3.3.1 Access to fertility centres providing standardised treatment

**Figure 3:**
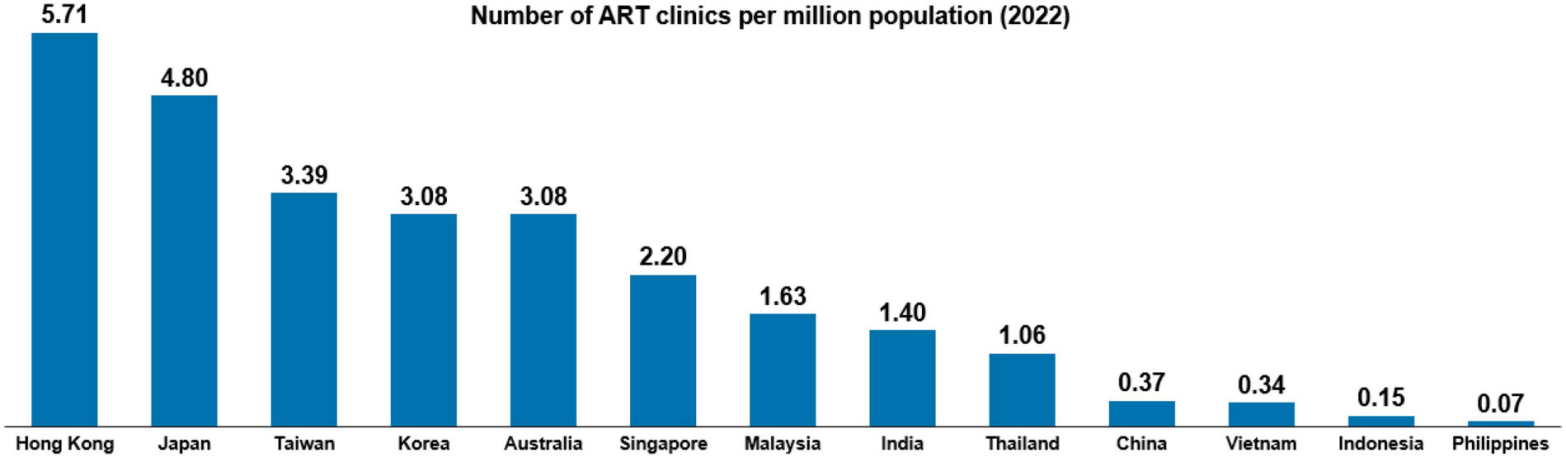
Number of ART clinics per million population (2022). International federation of fertility Societies’ surveillance (IFFS) 2022 (21); World Bank 2022 (78).

Extent of access to ART treatment is firstly informed by the number of fertility centres. In the APAC region, the limited number of such centres, located primarily in urban areas, poses a unique challenge as many countries and territories in the region have large populations with a wide geographical spread. As a result, there are often large rural populations that are partially disconnected from urban centres; this includes China, Indonesia, Malaysia, and the Philippines. Figure 3 demonstrates that these countries report a comparatively low ratio of ART clinics per million of population, highlighting potentially insufficient service provision. Literature echoes this; a study from China reported that over 25% of the population had no access to an ART clinic in their city. Authors found that most ART clinics are in the East of China, with Western cities remaining largely underserved (79). Similarly, during the Policy Roundtable discussion, experts highlighted that in relatively (based on population) smaller countries such as the Philippines and Malaysia, patients from rural areas may face difficulties in accessing a centre.

Geographic proximity is particularly consequential given the intensive and time-dependent nature of infertility treatment pathways. ART typically involves multiple stages, clinical consultation, diagnostic assessments, controlled ovarian stimulation, oocyte retrieval, and embryo transfer, requiring frequent interactions with specialised facilities over a relatively short period. A standard IVF cycle entails six to eight clinic visits within approximately six weeks, with additional visits often required across multiple cycles depending on patient characteristics and treatment response (80). For patients residing far from ART centres, this repeated-visit model imposes significant logistical, financial, and psychological burdens, which may affect both the initiation of treatment and the ability to persist with care.

Infertility treatment frequently requires more than one cycle to achieve a pregnancy, meaning that effective access depends on sustained engagement rather than one-time contact with services. A 2022 study from Australia found that women living within 15 kilometres of an IVF clinic were more likely to pursue treatment and consider going through additional IVF cycles in case of failure (80). Distance-related costs, such as travel, accommodation, and time away from work, may exacerbate these pressures for patients living outside urban centres, particularly in the absence of supporting infrastructure or subsidies. As a result, geographic barriers may translate into earlier discontinuation and reduced cumulative chances of pregnancy, even where ART services are technically available.

In addition to spatial and logistical factors, regulatory consistency and quality assurance play a critical role in shaping effective access to ART in the region. While regional professional bodies such as the Asia Pacific Initiative on Reproduction (ASPIRE) have developed minimum laboratory and clinical standards tailored to low- and middle-resource settings, implementation and enforcement vary substantially across jurisdictions (81). Weak or uneven regulation can affect treatment quality, patient confidence, and clinical outcomes, particularly in contexts where ART provision has expanded rapidly. In India, for instance, emerging evidence highlights ongoing concerns regarding regulatory capacity, standardisation, and oversight in ART provision despite recent legislative reforms, raising questions about the consistency and reliability of care delivered across centres (82).

Taken together, these factors indicate that access to ART in the APAC is shaped by a convergence of supply-side constraints and system-level characteristics. The concentration of clinics in urban areas limits geographic reach; the intensive nature of ART care pathways magnifies the burden of distance; and uneven regulatory environments affect both quality of care and patient trust. Addressing access disparities therefore requires attention to expanding the number of ART centres, and to their equitable distribution, integration within patient-centred care pathways, and regulation through robust and enforceable quality standards.

#### 3.3.2 Funding for ART services

A central policy challenge affecting access to ART treatment across the APAC region is the absence of comprehensive public funding and reimbursement pathways in several countries and territories. As shown in Table 4, India, Indonesia, the Philippines, Thailand, and Vietnam, do not provide national-level public reimbursement for ART treatment, leaving fertility care largely financed through direct out-of-pocket payments. Even in countries and territories where some public support exists, funding mechanisms vary widely in scope and eligibility criteria, reflecting fragmented policy approaches rather than a consistent health-system response to address infertility.

**Table 4:** Public funding availability for ART treatments.

Country/territory	Funding mechanism	Eligibility criteria for funding	Covered treatments
Australia (83)	Partial public funding through Medicare	Rebates apply universally	Clinically indicated ART, including IVF, intracytoplasmic sperm injection (ICSI), intrauterine insemination (IUI), embryo transfer, and associated consultations and diagnostics
China (84)	Partial public insurance coverage plus local government subsidies (varies by province/city)	Region-specific eligibility criteria: marital status requirements, documented infertility diagnosis, and age thresholds	IVF, IUI, ovulation induction and related procedures; reimbursement and eligibility vary by region; commonly reimbursed up to 2 cycles
Hong Kong (85)	Public provision via subsidised public hospitals	Married heterosexual couples; residency requirements and public hospital conditions may apply (e.g., some hospitals apply an age cap of 40)	IVF and related procedures
India (82)	No public funding		
Indonesia (42)	No public funding		
Japan (86)^a^	National public health insurance, supplemented by local subsidies	Age thresholds (<43 years of age), marital status, and clinical diagnostic criteria for infertility	IVF, ICSI, IUI and related treatments; 3–6 cycles depending on age
Korea (87)^a^	National Health Insurance plus local government subsidies	Age thresholds (e.g., fresh IVF is supported up to age 44), income-based subsidy tiers, marital status requirements	IVF, IUI, embryo freezing and related procedures; caps on reimbursable cycles
Malaysia (88)^a^	Partial public subsidies through government and university hospitals	Eligibility determined at institutional level	IVF and IUI through public and university hospitals
Philippines (71)^a^	No public funding		
Singapore (89)^a^	Direct government co-funding at public hospitals	Married heterosexual couples only; applies only at public hospitals; age thresholds (<40 years of age)	IVF, ICSI, frozen embryo transfer and IUI; 3 cycles are covered
Taiwan (90)^a^	Direct national subsidy programme	Married couples only; income-based criteria; subsidies are tiered based on age	IVF; caps on total subsidised cycles (up to 6, depending on eligibility)
Thailand (91)^a^	No public funding (policy proposals under discussion)		
Vietnam (92)^a^	No public funding		

^a^ Confirmed with experts during Policy Roundtable.

The lack of public funding presents a significant challenge in the APAC region because it intersects with high levels of income inequality and limited insurance coverage of fertility care. According to the United Nations Development Programme (UNDP), countries such as China, India, and the Philippines rank among those with the highest income inequality in the region (93). In these settings, reliance on private provision means that access to ART is closely tied to household financial capacity rather than clinical need.

The financial burden of ART treatment is substantial. Evidence from South-East Asia indicates that the direct out-of-pocket cost of a single ART cycle ranges from approximately US$1,000 to US$5,596, levels that may be prohibitive for large segments of the population (20). Importantly, ART treatment costs in the region have been estimated at approximately twice the average GDP per capita, underscoring the extent to which fertility care is misaligned with average income levels (20). In Vietnam, for example, patients have been reported to pay more than 100% of their annual income for a single ART cycle, illustrating the severity of access barriers in the absence of public financial protection (20). These affordability constraints are particularly acute in countries without public reimbursement mechanisms (Table 4) and where ART is delivered almost exclusively through the private sector (81).

The combined effect of limited public funding and high out-of-pocket costs is reflected in substantially lower utilisation of ART services in countries without reimbursement mechanisms. As illustrated in Figure 3, countries with limited or no public funding for ART, such as India and Indonesia, report very low numbers of ART treatment cycles per million population compared with countries that offer public reimbursement or subsidies, such as Japan, Australia, and Taiwan. The Asia-wide average similarly remains well below levels observed in countries with established public funding frameworks, indicating constrained uptake at the regional level.

These utilisation patterns suggest that ART treatment is systematically under-used in settings where patients must self-finance care, not because of lower clinical need, but because of financial barriers. In countries and territories without public funding, namely India, Indonesia, the Philippines, Thailand, and Vietnam (Table 4), ART treatment is therefore effectively inaccessible for many patients, particularly those from lower-income households. This reliance on private finance reinforces existing socioeconomic inequalities in reproductive health and limits the ability of large segments of the population to pursue parenthood through medical assistance (81).

Even in countries and territories where public funding is available, restrictive eligibility criteria further constrain effective access. Reimbursement policies frequently impose age limits, marital status requirements, and caps on the number of funded cycles, narrowing the population eligible for support. For example, in Taiwan, where relatively generous national subsidies are available, only married couples are eligible for public reimbursement, excluding single women and unmarried couples from funded care (94, 95). In addition, low caps on the number of reimbursed cycles, often without clear clinical justification, may reduce the likelihood that eligible patients can complete the number of cycles commonly required to achieve a successful pregnancy.

Overall, evidence from across the APAC region indicates that public funding design, and its absence, has a measurable impact on ART utilisation and access. Where comprehensive funding is lacking, treatment uptake remains low, as reflected in the number of ART cycles performed (Figure 2); where funding exists, restrictive eligibility criteria continue to limit who can benefit. Together, these policy features constrain patient access to ART and perpetuate socioeconomic inequities in fertility care across the region.

### 3.4 Psychosocial support

Infertility and associated treatments have significant effects on the physical and mental well-being of an affected woman (96). Additionally, due to cultural nuances, women and couples in Asia are often under social pressure to build a family. According to the literature, patients going through egg freezing or ART treatment are likely to develop psychiatric disorders such as anxiety and depression, as a result of stress (97). For instance, people with infertility in China have a higher likelihood of depression, with the prevalence of depressive symptoms ranging between 14% and 50%, compared to a prevalence of 2.4% in the general Chinese population (98).

The lack of availability of psychosocial support for infertility patients was identified as a significant challenge across most countries and territories in our sample. Key factors include a shortage of trained HCPs, insufficient psychosocial care providers, and a lack of national guidelines for psychological care in fertility settings (99). Studies show that in China, despite ongoing developments of mental health services, there are a limited number of HCPs trained in counselling in general (98), which likely means that there is a shortage of counsellors for patients with infertility. Using the existence of professional organisations as a proxy for the existence of trained HCPs and national guidelines, only Australia and Japan have national infertility counselling organisations (100). During the Policy Roundtable, experts highlighted that psychosocial support is often deprioritised by clinics, and patients must seek such services separately, resulting in a fragmented pathway. Additionally, experts emphasised, that support offered outside of clinics may not be sufficiently specialised to address the concerns and challenges of patients affected by infertility, which may result in suboptimal outcomes.

Psychosocial counselling for patients experiencing infertility is not reimbursed or subsidised in most APAC countries and territories (39). Since ART treatments are often not reimbursed, and frequently exceed the annual income of patients, individuals may choose to forgo fertility counselling, instead opting to use any financial bandwidth they have for an additional ART cycle. However, the resulting stress can negatively affect patients' mental health and may lead to discontinuation of treatment.

### 3.5 Use of supplementary care

We use the term “supplementary care” to refer to additional services that are offered alongside standard ART treatments to enhance the chances of conception and successful pregnancy. We consider two distinct types of supplementary care:

- **Preimplantation genetic testing:** PGT is a set of tools for prenatal diagnosis to identify chromosomal abnormalities and avoid the implantation of affected embryos. The goal of PGT is to prevent the transmission of pathologic genetic conditions and hence to improve outcomes of fertility treatment. In this research, we focus on PGT-M (testing for genetically transferable conditions) and PGT-SR (testing for chromosomal structural rearrangements) (101, 102).
- **Non-validated “add-ons”:** There are also optional treatments and procedures that often come with claims that they can improve fertility outcomes; however, they may lack robust clinical evidence to support this. Common add-ons include endometrial scratching, assisted hatching of embryos, and complementary therapies such as acupuncture (103). Because no high-quality, robust clinical trials confirm the value of such treatments, their efficacy and safety profiles are unknown (104). We refer to such treatments as non-validated add-ons.

Our assessment looked for policies that either support access to clinically validated supplementary care or regulate access to non-validated supplementary care.

Availability of PGT to ensure the health of the embryo is important for individuals going through ART treatment. Studies have shown that the critical motivation to pursue PGT is to have a “biologically related child at significantly reduced risk of inheriting a genetic condition” (105). Nonetheless, in some countries and territories, PGT is only available to couples with known hereditary conditions, creating barriers for those who may benefit from testing but do not have a documented family history of genetic disorders. While this restriction is based on ethical guidelines, it may limit patients' ability to pursue a healthy pregnancy. In some countries and territories, PGT is not offered across all fertility clinics due to cultural and religious reasons; an example of this is the Philippines (106). This inconsistency in service availability can lead to disparities in access to advanced reproductive technologies, leaving many patients without options that could enhance their chances of a successful and healthy pregnancy.

In many APAC countries and territories, the absence of reimbursement for PGT means that patients must cover the costs entirely, which can be expensive. As shown in Table 5, public funding for PGT-M and PGT-SR is rare across the region. When patients must pay out of pocket for both ART treatment and PGT, fertility care becomes unaffordable for most patients. As a result, those who would benefit from PGT—such as couples with a family history of genetic disorders—may choose to forgo this essential service due to financial constraints. This not only limits access to potentially life-saving information but can also lead to increased emotional stress for patients who are already navigating the complexities of infertility. For instance, a study from Malaysia, where there is limited availability of funding options, found that many respondents consider PGT services very costly, which disincentivises them from seeking them (124).

**Table 5:** Availability and public funding of PGT.

Country/territory	PGT-M/PGT-SR: availability^a^	PGT public funding
Australia	Available (107)	Partial (108)
China	Available (109)	Limited/local (varies by province) (110)
Hong Kong	Available (111)	No (111)
India	Available (112)	No (112)
Indonesia	Available (113)	No (113)
Japan	Available (114, 115)	No^b^ (116)
Korea	Available across major fertility centres (117)	No^b^ (118)
Malaysia	Available (119)	No^b^
Philippines	Limited availability; not offered across all clinics due to cultural and religious factors (120)	No^b^ (120)
Singapore	Available (72)	Partial^b^ (72)
Taiwan	Available (121)	No^b^ (121)
Thailand	Available (122)	No^b^
Vietnam	Available (123)	No^b^

^a^ Summarises whether such services are legally permissible in the countries and territories in scope.

^b^ Confirmed with experts during Policy Roundtable.

Patients across the APAC region may opt to purchase non-validated add-ons to their fertility treatments, which come with additional costs but limited demonstrated efficacy (38). A study in Australia found that 72% of add-ons incurred an additional cost to the patient receiving ART treatment (103). Such additional interventions are typically not subject to rigorous regulation or scientific validation; studies report that none of the most used add-ons are supported by high-quality evidence, and there are frequent inconsistencies between the perceived safety of add-ons and evidence (103, 125). As a result, patients may invest significant financial resources in these treatments without a clear understanding of their suitability, and basing their choice on false perceptions of effectiveness. While there is a lack of literature assessing the regulation of non-validated treatments, during the Policy Roundtable, experts highlighted that such services are usually governed by hospital- or clinic-specific guidance, rather than strict laws.

### 3.6 Scoring the level of policy development

Under each category, we qualitatively scored the level of policy development in each in-scope APAC country and territory to identify where future policy development is most needed. In these scores, red indicates a general absence of policy action and funding, while amber indicates that some positive progress has been made, but there are still significant shortcomings impacting patient access and care, and green indicates a well-developed policy environment that is supportive of patient access and care. These scores are provided for directional purposes only, with the intent to guide discussions at a national level on how efforts and public resources can be allocated in the most impactful manner.

**Figure 4:**
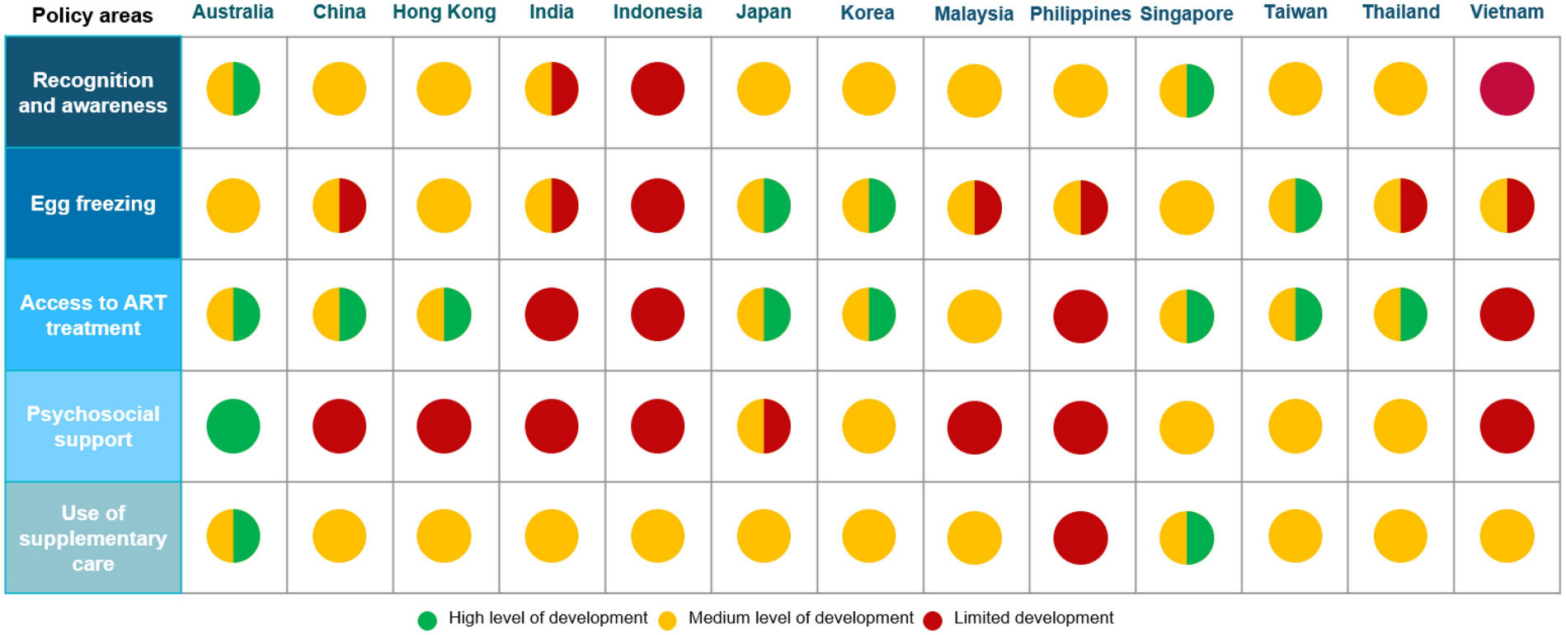
Qualitative scoring of fertility policy development across Asia Pacific countries. High level of development is defined as having evidence of political prioritization of fertility (for example through published plans or strategies) and existence of regulations and public funding to support access to egg freezing, fertility treatment, psychosocial support and validated supplementary care. A medium development reflects some evidence of political prioritization and willingness, but ineffective implementation of policies or insufficient availability of public funding. A low development is defined as the absence of evidence of political prioritization and the absence of public funding to support patient access and care. Source: CRA analysis.

The results of the qualitative scoring are shown in Figure 4. Several patterns emerge:

- There is a large amount of variation across countries in the extent to which policy has been developed to support patients seeking infertility treatment and care. This suggests that a tailored approach to policy improvement is needed.
- At the same time, there are some common underserved policy areas across the region: reading down the rows in Figure 4, patients across all countries and territories experience challenges related to the recognition and awareness of infertility and access to psychosocial support.
- There appears to be a trickle-down effect: political recognition of infertility as a medical condition (and hence a condition eligible for support and coverage under health budgets) is absent or weak in many countries and territories. We see in Figure 4 that this often results in a lack of reimbursement for egg freezing or fertility treatment, limiting patient access to care.

## 4 Discussion: future goals for optimising patient access and care

Building from the policy gaps identified, we have developed recommendations for each policy area, aiming to establish recognition of infertility as a disease, improve patient access to relevant services, and ensure there is support available across the patient journey to ensure maximum chance of successful outcomes.

### 4.1 Policy recommendations on recognition and awareness of infertility

A central challenge identified during the Policy Roundtable is the persistent lack of formal recognition of infertility as a disease, which contributes to misconceptions that it stems largely from individual or lifestyle choices. Existing global guidance, most notably the WHO's 2009 statement classifying infertility as a disease of the reproductive system (126), has long been emphasised in academic and policy literature as a critical foundation for destigmatising infertility and improving access to care. Despite this, adoption of this classification across APAC remains inconsistent, and infertility continues to impose a significant disease burden throughout the region (Figure 1). Because limited formal recognition constrains policy prioritisation and investment, experts from the Policy Roundtable recommended that APAC governments formally recognise infertility as a disease and develop national plans to address challenges including access to medical egg freezing and ART.

A related challenge concerns low levels of fertility awareness among the public, particularly regarding the relationship between age and infertility. A substantial body of research demonstrates that structured, early education increases awareness of age-related fertility decline and enables individuals to make informed reproductive decisions. Australia, often cited for its comparatively strong fertility policy environment, illustrates this point: it has the highest global proportion of children born through ART (5%) (127), and recent reports indicate that over half of Australian women believe they have strong fertility awareness and perceive ART as accessible (128). However, such approaches are uneven across APAC, and young people often lack formal opportunities to learn about infertility risks. To address this, experts from the Policy Roundtable recommend embedding information on infertility and its connection to age within formal sexual health education in schools to better prepare young people as they transition into adulthood.

Another challenge is the limited availability of accessible, culturally relevant information on infertility and treatment options, which contributes to delays in care and suboptimal help-seeking behaviour. Studies show that delivering reproductive health education and social behaviour change messages in local languages and culturally relevant formats enhances knowledge and awareness, supporting increased uptake of services (129). Yet across APAC, such initiatives remain fragmented or insufficiently scaled to reach affected populations. In response to these limitations, experts from the Policy Roundtable propose implementing targeted education campaigns and community-based outreach in local languages to strengthen public awareness of available infertility treatments.

### 4.2 Policy recommendations on egg freezing

As outlined earlier, women may pursue egg freezing for various reasons, including to safeguard fertility before undergoing fertility-damaging treatments like radiation or chemotherapy. However, beyond medical reasons, many women also opt for egg freezing for social reasons, such as delaying childbirth to focus on career development or personal circumstances. Growing demand underscores the need for more widespread access to egg freezing, which is strongly supported by experts in the region.

#### 4.2.1 Medical egg freezing

Across APAC health systems, studies and experts report that women undergoing fertility-compromising treatments receive insufficient or delayed information about medical egg freezing, limiting their ability to pursue fertility preservation within the necessary clinical timeframe. Studies in multiple Asian countries show that fertility preservation services are still developing and that low awareness among healthcare providers, lack of established referral pathways, and insufficient multidisciplinary collaboration hinder timely counselling and referral to reproductive specialists (130, 131). International guidelines, such as those issued by the American Society of Clinical Oncology (ASCO), emphasise the importance of early counselling and timely referral to fertility specialists, recommendations that are equally applicable in APAC-specific contexts (132, 133). However, existing guidance does not adequately address persistent gaps in HCP training, culturally sensitive communication, and infrastructural variability that continue to delay referrals (134). During the expert Policy Roundtable, participants highlighted that educational initiatives in the APAC region should include tailored training modules for oncology and gynaecology HCPs, accompanied by clear referral pathways and culturally appropriate counselling tools. Such measures would help ensure that medical egg freezing is consistently recognised as a time-critical intervention and that eligible patients are referred without unnecessary delay.

Access, however, is also shaped by affordability. As discussed in Section 3.2, high out-of-pocket costs remain a significant deterrent, leading some patients to forgo fertility preservation altogether. This financial barrier is particularly striking in light of evidence demonstrating substantial public support for funding. An online survey of 656 women in Australia, found that 87% supported some form of public funding for medical egg freezing, with 46% endorsing full coverage through the public health system (135). The authors suggest that these findings should inform policy review regarding the funding of egg freezing, highlighting a disconnect between current cost burdens and public attitudes toward reimbursement. This position was echoed by experts at the Policy Roundtable, who emphasised that medical egg freezing should be supported through both public funding mechanisms and private insurance coverage to promote equitable access across healthcare settings.

#### 4.2.2 Social egg freezing

Women continue to face substantial barriers that limit their ability to make informed and timely decisions about fertility preservation. Legal restrictions, cultural norms, limited healthcare integration, and high out-of-pocket costs collectively constrain access to egg freezing services. Legalising social egg freezing is therefore a positive step towards expanding reproductive autonomy in the region. Recent literature supports this position, arguing that access to social egg freezing should not be restricted to medical indications or marital status, but made available to women who desire it (136). Such analyses frame the procedure as a legitimate means of preserving future reproductive choice in societies where structural and cultural factors often delay family formation.

Due to overall limited awareness of infertility, many women lack timely and accurate information about their fertility potential, which limits their ability to make informed decisions about egg freezing. Expert insights from the Policy Roundtable highlight the value of incorporating ovarian reserve testing, particularly anti-Müllerian hormone (AMH) testing, as an early indicator of reproductive potential, enabling women to understand whether egg freezing is appropriate for them. This approach is already reflected in emerging policy practice; for example, Korea has introduced reimbursement schemes that use AMH levels as one of the criteria for subsidising egg freezing, illustrating how fertility testing can be operationalised within funding frameworks to guide timely decision-making (137). While these efforts demonstrate the feasibility and benefits of integrating fertility diagnostics into policy, such practices remain limited across most APAC jurisdictions. To improve awareness and support earlier engagement with fertility preservation, policymakers should standardise the proactive offer of fertility testing, such as AMH testing, as part of routine care for women of reproductive age, ensuring that decisions about egg freezing are informed by clear clinical guidance. Furthermore, such efforts should be coupled with the widespread provision of information on when best to seek out egg freezing, helping ensure women receive practical, age-specific guidance on optimal timing and are equipped to make informed choices that maximise future reproductive outcomes.

Finally, as explored in Section 3.2, access to social egg freezing is largely shaped by the availability of public funding mechanisms that support its affordability. Only three countries and territories included in the scope of this study provide public funding support for social egg freezing, highlighting that the financial barriers remain one of the most significant obstacles to equitable access. Although literature acknowledges these economic challenges, current government funding models in the APAC region remain insufficient. To improve equitable access and support long-term reproductive planning, governments should introduce dedicated public funding or subsidy schemes for social egg freezing. Expert Policy Roundtable discussions highlighted that women should have access to and reimbursement of egg freezing regardless of reason. This sentiment is further supported in literature; according to a recent survey conducted among APAC-based fertility experts, almost 90% support better availability of egg freezing (both medical and social) (136). Japan is an example of a country that has been developing policies to support women hoping to preserve their fertility potential. Specifically, the Tokyo Metropolitan Government offers ¥300,000 (US$2,000) for egg freezing to women between the ages of 18 and 29 wanting to delay childbirth (138). Since the subsidy was introduced, more than 9,000 women have expressed interest in this service, demonstrating a growing demand for egg freezing in Tokyo (138). According to recent media reports, Tokyo policymakers are planning to increase the allocated budget to meet the increasing demand (138). This is further strengthened by information sessions that provide educational materials to women exploring egg freezing, ensuring they are well-informed about their options.

### 4.3 Policy recommendations on access to treatment

While some countries and territories in the APAC region have made significant advances in improving access to ART treatment, others are only beginning this journey, and treatment therefore remains inaccessible to many. During the Policy Roundtable, experts reached a consensus on the importance of ensuring that each country and territory develops the necessary infrastructure to make fertility care easily accessible. Specifically, this entails improving availability of fertility clinics, covering both urban and rural areas to ensure that such clinics can meet the demands of the population. While similar research is lacking for the APAC region, a study from Portugal highlights that tailored policy interventions that address sociodemographic barriers are vital to ensure equity, which is certainly relevant for countries and territories in scope (139). Experts from the Policy Roundtable therefore recommend expanding the number and geographic distribution of fertility centres to support equitable access to ART services across diverse populations.

Financial barriers also significantly restrict access to treatment. Survey evidence shows that most fertility specialists in the region believe government-supported funding is essential to make ART accessible (136). Current policies, however, often exclude key groups, such as couples who have previously undergone ART cycles or women over 40, leaving many without affordable options despite medical need. To improve affordability and reduce inequities, experts from the Policy Roundtable recommend increasing government investment in ART and ensuring funding schemes are broad enough to include all patients requiring treatment, irrespective of age or treatment history.

Policy design plays a crucial role in determining who can use ART services. Taiwan's experience illustrates the importance of adjusting reimbursement criteria to better reflect real-world needs. Before 2021, subsidies were limited to low-income married couples, restricted to one cycle per year, and applicable in only 20 clinics (90), resulting in extremely low uptake of 12–20 cases annually (140). Following policy expansion, to include all married couples, increase the number of cycles covered, and extend coverage to 101 clinics (90), uptake rose sharply, with 18,157 couples receiving subsidies and 20,539 babies born via IVF by July 2024 (140). Broader economic analyses also show strong fiscal justification for ART funding: in Australia, ART reimbursement has long yielded positive returns, with each US$1 spent in 2021 projected to generate three times that amount in future tax revenue (141), and multiple IVF cycles shown to be cost-beneficial for women under 42 (142, 143). Reflecting this evidence, experts from the Policy Roundtable recommend revising reimbursement frameworks to make eligibility criteria more inclusive and thereby improve equitable access to ART across the region.

### 4.4 Policy recommendations on provision of psychosocial support

Patients experiencing infertility frequently face significant emotional and psychological burden, including stress, anxiety, and uncertainty about treatment outcomes and future family-building. Research consistently shows that emotional support from trained HCPs is highly valued by patients undergoing fertility treatment (144), and such support reduces treatment discontinuation while improving the overall treatment experience, as discussed in Section 3.4. Despite this evidence, access to adequately trained HCPs remains uneven across APAC, and psychosocial care is not always regarded as a core component of fertility services. Experts from the Policy Roundtable therefore recommend expanding training opportunities for HCP to equip them with the skills required to deliver high-quality psychosocial support and to increase the number of qualified providers available to patients undergoing ART.

Beyond workforce capacity, the integration of psychosocial care into fertility treatment pathways remains inconsistent, leaving many patients without structured support at critical moments during ART. Literature indicates that embedding psychosocial services throughout treatment helps patients process emotions, manage expectations, and cope with uncertainty (144). Policymakers in Victoria, Australia, offer an illustrative example: fertility counselling is made available both prior to and throughout ART treatment, forming part of a clearly defined patient pathway (145). This model demonstrates how routine integration of counselling can normalise support-seeking and assist individuals in navigating the complex emotional landscape of infertility. Reflecting this evidence, experts from the Policy Roundtable recommend formally embedding psychosocial care at key treatment milestones and offering access whenever requested by the patient, ensuring that support is proactive rather than optional or *ad hoc*.

Affordability is another barrier preventing many individuals from accessing psychosocial care during fertility treatment. Victoria again provides a relevant example: counselling services are subsidised by the state and available across participating public clinics (145), and this practice has influenced broader adoption across Australia. Many private clinics, such as IVFAustralia, now also offer psychosocial support free of charge, both in person and via telephone consultations (146). These models show how financial support can widen access and ensure that psychosocial care is not restricted to those with the ability to pay. However, similar funding mechanisms are not consistently available across APAC, resulting in inequities in emotional support provision. Experts from the Policy Roundtable therefore recommend that governments provide dedicated funding for psychosocial services, such as fertility counselling, to ensure equitable access for all individuals undergoing ART.

### 4.5 Policy recommendations regarding the use of supplementary care

As outlined in Section 3.5, many individuals undergoing ART seek supplementary interventions to increase the likelihood of a successful outcome, including PGT for inherited diseases. Evidence shows that testing for monogenic or structural chromosomal abnormalities (PGT-M and PGT-SR) can help patients with a familial history of conditions such as cystic fibrosis or sickle cell disease to understand whether their embryos have inherited disease-causing variants. Access to these forms of PGT is tightly regulated in many settings due to ethical considerations, yet they remain important tools for reducing uncertainty regarding embryo health. Singapore offers a relevant example: both PGT-M and PGT-SR are available in public hospitals, but clinics must first receive Ministry of Health approval, and eligible couples can access government co-funding (89). Despite these models, availability across APAC remains uneven. Experts from the Policy Roundtable therefore recommend that PGT for relevant familial disease histories be made accessible to eligible patients and reimbursed to improve equitable access and reduce uncertainty regarding embryo health.

Alongside clinically validated interventions such as PGT-M and PGT-SR, patients frequently express interest in add-on treatments that lack strong evidence of benefit. The desire for such add-ons is often driven by uncertainty, emotional burden, or the hope of increasing treatment success. Yet research consistently highlights that non-validated add-ons rarely improve outcomes and may expose patients to unnecessary financial or physical burdens. Currently, patient information on the efficacy of these interventions is inconsistent across APAC, contributing to misunderstandings and unrealistic expectations. Experts from the Policy Roundtable therefore recommend proactive dissemination of clear, evidence-based information on non-validated add-ons to help patients make informed decisions about their care.

Regulatory oversight of non-validated add-ons also varies across the region. While this research did not identify infertility-specific regulations governing add-on provision in APAC, broader health regulations are sometimes applicable. For example, Korea enforces strict laws governing the advertisement of medical procedures, including add-ons offered in fertility care (147). However, in most APAC countries and territories, add-ons with limited evidence-based efficacy remain insufficiently regulated, allowing potentially misleading claims or inappropriate use. Reflecting these gaps, experts from the Policy Roundtable recommend strengthening regulation of non-validated add-ons across private and public clinics to ensure that only interventions with demonstrated benefit are incorporated into fertility treatment pathways.

### 4.6 Tailoring the recommendations to different policy environments

As our research identified that the policy environment is highly variable across the APAC region, we refer to the classification established in Section 3.6 to link to tailored recommendations for each level of policy development. The aim is that this can be utilised on a local level to prioritise and develop targeted policy strategies that reflect the most pressing challenges in each country and territory. A mapping of the recommendations to different types of policy environment is shown in Table 6.

**Table 6:** Policy recommendations stratified by current level of policy development.

Policy theme	Current level of policy development		
	Low	Medium	High
Recognition and awareness	Recognise infertility as a disease and draft national plans to address associated challenges such as access to medical egg freezing and ART treatment.	Include the concept of infertility and its connection to age in formal sexual health education in schools to ensure that young people are well-informed as they transition into adulthood.	Develop targeted education campaigns and events to ensure that the public and those affected by infertility have a good understanding of optimal treatment options.
Egg freezing	In educational campaigns, include support for HCP awareness of medical egg freezing for patients going through fertility-compromising treatment to ensure referral to the appropriate specialists in time. Make funding available for patients seeking medical egg freezing, both publicly and through insurance in private hospitals. Legally permit social egg freezing for all patients who desire it.	Proactively offer fertility testing to women during their reproductive years to inform them of their fertility potential and discuss egg freezing options.	Launch educational campaigns to improve general awareness on when best to seek out egg freezing services to ensure optimal outcomes. Provide government funding for social egg freezing to improve access to services.
Access to ART treatment	Open more fertility centres, particularly in rural areas, to support equity of outcomes.	Increase government funding to support affordability of ART treatments.	Where funding is already available for ART treatment, make reimbursement criteria more inclusive to allow equitable access to services and improved affordability.
Psychosocial support	Provide more training opportunities for HCPs to offer psychosocial support, and thereby, increase the number of qualified HCPs to offer this service.	Ingrain psychosocial care into the patient pathway as they begin ART treatment at key milestones during treatment and as requested by the patient.	Government provides funding for psychosocial care such as fertility counselling to support equitable patient access during ART treatment.
Use of supplementary care	Make PGT available for patients with relevant familial history of disease to increase successful outcomes and dispel uncertainties over the health of the child.	Reimburse PGT for patients with relevant familial history of disease. Proactively share available information on non-validated add-ons and their efficacy with patients, to increase overall awareness of their impact on ART treatment outcomes.	Ensure that non-validated add-ons with limited evidence-based efficacy are well-regulated in both private and public clinics.

High level of development is defined as having evidence of political prioritization of fertility (for example through published plans or strategies) and existence of regulations and public funding to support access to egg freezing, fertility treatment, psychosocial support and validated supplementary care. A medium development reflects some evidence of political prioritization and willingness, but ineffective implementation of policies or insufficient availability of public funding. A low development is defined as the absence of evidence of political prioritization and the absence of public funding to support patient access and care.

CRA analysis.

The outlined recommendations are meant to act as forward-looking goals for the countries and territories in scope. Nonetheless, it is vital to acknowledge that implementation of the recommendations will require careful consideration of the economic, social, and cultural contexts of the countries and territories, as these may act as hurdles. Policymakers should collaborate with academic, clinical, and industry stakeholders to develop and enforce optimal fertility policies that maximise patient access.

## 5 Conclusion and avenues for further research

This review has examined the key challenges that impact the treatment pathway of individuals with infertility. Through an extensive literature review and an engaged discussion with leading APAC fertility experts, we identified a range of policy challenges across the APAC infertility treatment landscape. The proposed policy recommendations aim to address these challenges by advocating for the recognition of infertility as a disease, establishing infrastructure for ART treatment provision, and expanding public funding. Looking to the history of policy development and TFR decline in Korea as an example, it is important that policy action is proactive rather than reactive in response to TFR declining below the replacement rate.

Future research should focus on assessing the long-term impact of policy changes on access, equity, and treatment outcomes, as well as incorporating patient experiences and quality-of-life measures to better inform policy development. Additionally, views of policymakers should be captured in a similar manner.

## 6 Limitations

This study has several limitations. The literature review relied on publicly available sources, which vary in depth and completeness across APAC countries and may not fully capture emerging or unpublished policy developments. Expert validation, while valuable, involved a limited number of participants from nine countries and territories, and their insights may reflect individual professional backgrounds or institutional perspectives. The expert engagements, though informative, provided constrained time for exploring complex policy areas, and group dynamics may have influenced which viewpoints were expressed. This study aimed to capture forward-looking recommendations, nonetheless, future research should explore operational considerations on how the recommendations can be implemented in practice. Finally, the study is qualitative in nature and does not assess policy outcomes or establish causal relationships, meaning the findings should be interpreted as a synthesis of existing evidence and expert perspectives rather than a comprehensive empirical evaluation.

## References

[1] Kato H. Declining population and the revitalization of local regions in Japan. Meiji J Political Sci Econ. (2014) 3:25–35.

[2] Bhattacharjee NV Schumacher AE Aali A Abate YH Abbasgholizadeh R Abbasian M Global fertility in 204 countries and territories, 1950–2021, with forecasts to 2100: a comprehensive demographic analysis for the global burden of disease study 2021. Lancet. (2024) 403:2057–99. 10.1016/S0140-6736(24)00550-6 38521087 PMC11122687

[3] Tsuya N. The impacts of population decline in Japan: demographic prospects and policy implications. Age. (2014) 15:64.

[4] Liu Y Westelius N. The impact of demographics on productivity and inflation in Japan. J Int Commer Econ Policy. (2017) 8(02):1750008. 10.1142/S1793993317500089

[5] Economist Impact. (2023). Fertility policy and practice: a Toolkit for the Asia-Pacific region. Available online at: https://www.fertilitycounts.com/wp-content/uploads/2023/05/Economist_Impact_Fertility_Policy_Toolkit.pdf (Accessed January 20, 2026).

[6] World Health Organization. (2023). Infertility prevalence estimates 1990–2021. Available online at: https://www.who.int/publications/i/item/978920068315

[7] Luo Y Hong C Fan H Huang Y Zhong P Zhao Y Trends and distribution of infertility—Asia Pacific region, 1990–2021. China CDC Wkly. (2024) 6:689–94. 10.46234/ccdcw2024.155 39035872 PMC11255606

[8] Somigliana E Paffoni A Busnelli A Filippi F Pagliardini L Vigano P Age-related infertility and unexplained infertility: an intricate clinical dilemma. Hum Reprod. (2016) 31:1390–6. 10.1093/humrep/dew066 27060173

[9] Central Intelligence Agency. Field Listing: Mother’s mean age at first birth. Accessible at: Available online at: https://www.cia.gov/the-world-factbook/field/mothers-mean-age-at-first-birth/ (Accessed January 20, 2026).

[10] Gao Y Liu H Qiao L Liang J Yao H Lin X Study of burden in polycystic ovary syndrome at global, regional, and national levels from 1990 to 2019. Healthcare 11:562. 10.3390/healthcare11040562 PMC9957370 36833096

[11] Chen T-H Chang S-P Tsai C-F Juang K-D. Prevalence of depressive and anxiety disorders in an assisted reproductive technique clinic. Hum Reprod. (2004) 19:2313–8. 10.1093/humrep/deh414 15242992

[12] Bagade T Thapaliya K Breuer E Kamath R Li Z Sullivan E Investigating the association between infertility and psychological distress using Australian longitudinal study on Women’s health (ALSWH). Sci Rep. (2022) 12:10808. 10.1038/s41598-022-15064-2 35752691 PMC9233676

[13] Loke AY Yu P Hayter M. Experiences of sub-fertility among Chinese couples in Hong Kong: a qualitative study. J Clin Nurs. (2012) 21:504–12. 10.1111/j.1365-2702.2010.03632.x 21507092

[14] Wiersema NJ Drukker AJ Mai BT Giang HN Nguyen TN Lambalk CB. Consequences of infertility in developing countries: results of a questionnaire and interview survey in the South of Vietnam. J Transl Med. (2006) 4:54. 10.1186/1479-5876-4-54 17192178 PMC1766365

[15] Imai Y Endo M Kuroda K Tomooka K Ikemoto Y Sato S Risk factors for resignation from work after starting infertility treatment among Japanese women: Japan-female employment and mental health in assisted reproductive technology (J-FEMA) study. Occup Environ Med. (2021) 78:426–32. 10.1136/oemed-2020-106745 PMC8142458 33273052

[16] Zegers-Hochschild F Adamson GD de Mouzon J Ishihara O Mansour R Nygren K The international committee for monitoring assisted reproductive technology (ICMART) and the world health organization (WHO) revised glossary on ART terminology, 2009. Hum Reprod. (2009) 24:2683–7. 10.1093/humrep/dep343 19801627

[17] Kupka MS Chambers GM Dyer S Zegers-Hochschild F de Mouzon J Ishihara O International committee for monitoring assisted reproductive technology world report: assisted reproductive technology, 2015 and 2016. Fertil Steril. (2024) 122(5):875–93. 10.1016/j.fertnstert.2024.07.009 38996903

[18] Dyer S Chambers GM Adamson GD Banker M De Mouzon J Ishihara O ART Utilization: an indicator of access to infertility care. Reprod Biomed Online. (2020) 41:6–9. 10.1016/j.rbmo.2020.03.007 32448672

[19] Bauer A Goel A Faruqui D. (2023). Conceiving Success: Growth and Consolidation in IVF in APAC and the Middle East. Available online at: https://www.lek.com/insights/hea/sea/ei/conceiving-success-growth-and-consolidation-ivf-apac-and-middle-east (Accessed January 20, 2026).

[20] Njagi P Groot W Arsenijevic J Dyer S Mburu G Kiarie J. Financial costs of assisted reproductive technology for patients in low- and middle-income countries: a systematic review. Hum Reprod Open. (2023) 2023:hoad007. 10.1093/hropen/hoad007 36959890 PMC10029849

[21] Ory SJ Miller K Horton M. International federation of fertility Societies’ surveillance (IFFS) 2022: global trends in reproductive policy and practice, 9th edition. Glob Reprod Health. (2022) 7:e58. 10.1097/GRH.0000000000000058

[22] Adamson GD Armstrong H Cheong Y Damato E Fatemi H Ferriani R Policy solutions to improve access to fertility treatment and optimise patient care: consensus from an expert forum. Front Reprod Health. (2025) 7:1605480. 10.3389/frph.2025.1605480 40950655 PMC12426281

[23] Wisana IDGK Setyonaluri D. (2024). Indonesia’s Shifting Views on Marriage and Babies. Available online at: https://thediplomat.com/2024/09/indonesias-shifting-views-on-marriage-and-babies/ (Accessed January 20, 2026).

[24] Cheung E. (2024). Taiwan needs more babies. But conservative traditions are holding back some fertility solutions. *CNN*. Available online at: https://edition.cnn.com/2024/03/30/asia/taiwan-population-fertility-barriers-hnk-intl/index.html (Accessed January 20, 2026).

[25] Kane P Choi CY. China’s one child family policy. Br Med J. (1999) 319:992–4. 10.1136/bmj.319.7215.992 10514169 PMC1116810

[26] Wang GT. Population control policies and implementations in India. J Sociol Soc Work. (2019) 7:135–44. 10.15640/jssw.v7n2a14

[27] Permana IB Westoff CF. The two-child norm in Indonesia. Jakarta: National Family Planning Coordinating Board (1999).

[28] Dérer P. Thailand’s Success Story—family Planning with Creativity and Humor. The Overpopulation Project (2019). Available online at: https://overpopulation-project.com/thailand-success-story-family-planning-with-creativity-and-humor/ (Accessed January 20, 2026).

[29] Malaysia Population Research Hub. (n.d.). *Evolution of population policy*. Available online at: https://mprh.lppkn.gov.my/evolution-of-population-policy/ (Accessed January 20, 2026).

[30] Ngo AP. Effects of Vietnam’s two-child policy on fertility, son preference, and female labor supply. J Popul Econ. (2020) 33:751–94. 10.1007/s00148-019-00766-1

[31] Philippine Commission on Women. (2012). *Republic Act 10354: The Responsible Parenthood and Reproductive Health Act of* 2012. Available online at: https://pcw.gov.ph/republic-act-10354/ (Accessed January 20, 2026).

[32] Yun J Kim CY Son S-H Bae C-W Choi Y-S Chung S-H. Birth rate transition in the Republic of Korea: trends and prospects. J Korean Med Sci. (2022) 37:e304. 10.3346/jkms.2022.37.e304 36325608 PMC9623034

[33] Sharma A Kamboj N Saraswathy KN Puri M Babu N Mahajan C. Knowledge, attitude, and practice of infertility: a comparative study in infertile and fertile Indian women. J Biosoc Sci. (2023) 55:947–59. 10.1017/S0021932022000347 36189761

[34] UNESCO Office Bangkok, United Nations Population Fund, & International Planned Parenthood Federation. (2021). *Learn, protect, respect, empower: The status of comprehensive sexuality education in Asia and the Pacific; a summary review 2020*. Available online at: https://unesdoc.unesco.org/ark:/48223/pf0000377782 (Accessed January 20, 2026).

[35] Maeda E Sugimori H Nakamura F Kobayashi Y Green J Suka M A cross sectional study on fertility knowledge in Japan, measured with the Japanese version of cardiff fertility knowledge scale (CFKS-J). Reprod Health. (2015) 12:10. 10.1186/1742-4755-12-10 25638172 PMC4417216

[36] Shin H Lee J Kim SJ Jo M. Attitudes towards parenthood and fertility awareness in female and male university students in South Korea. Child Health Nurs Res. (2020) 26(3):329–37. 10.4094/chnr.2020.26.3.329 35004476 PMC8650971

[37] Sugimori H. Fertility knowledge and the timing of first childbearing: a cross-sectional study in Japan. Hum Fertil. (2016) 19:275–81. 10.1080/14647273.2016.1239033 27701914

[38] Patra S Unisa S. Addressing reproductive health knowledge, infertility and coping strategies among rural women in India. J Biosoc Sci. (2021) 53:557–65. 10.1017/S0021932020000371 32677598

[39] Ferring Pharmaceuticals. (2023). Real voices, new insights—Eureka moments for fertility in Asia. Available online at: https://ferring.sg/eureka/ (Accessed January 20, 2026).

[40] Chen WA Wu CL Ho HY Chang F Yang JH Kung FT Social determinants of health that impact the time to diagnosis and treatment of infertility in Taiwan. J Formos Med Assoc. (2024) 124:348–54. 10.1016/j.jfma.2024.05.002 38710607

[41] Domar A Vassena R Dixon M Costa M Vegni E Collura B Barriers and factors associated with significant delays to initial consultation and treatment for infertile patients and partners of infertile patients. Reprod Biomed Online. (2021) 43:1126–36. 10.1016/j.rbmo.2021.09.002 34756644

[42] Purvis TE. Assisted reproduction in Indonesia: policy reform in an islamic culture and developing nation. Reprod Biomed Online. (2015) 31:697–705. 10.1016/j.rbmo.2015.07.008 26371707

[43] Langston DM Fendereski K Halpern JA Aston KI Emery BR Ramsay JM Fertility outcomes across the rural-urban continuum. Fertil Steril. (2023) 120:e234–5. 10.1016/j.fertnstert.2023.08.664

[44] Ethics Committee of the American Society for Reproductive Medicine. Planned oocyte cryopreservation to preserve future reproductive potential: an ethics committee opinion. Fertil Steril. (2024) 121:604–12. 10.1016/j.fertnstert.2023.12.030 38430080

[45] Practice Committee of the American Society for Reproductive Medicine. Fertility preservation in patients undergoing gonadotoxic therapy or gonadectomy: a committee opinion. Fertil Steril. (2019) 112:1022–33. 10.1016/j.fertnstert.2019.09.013 31843073

[46] Abdul Karim AK Ahmad MF Abdul Hamid H. Fertility preservation opportunities for cancer patients in Malaysia. Med J Malaysia. (2021) 76:417–8. 34031343

[47] Chan LS Cochon KL Li TC Chung JPW Kim JH. Knowledge and intentions to use fertility preservation among urban Chinese cancer patients: a study from Hong Kong. PLoS One. (2024) 19:e0307715. 10.1371/journal.pone.0307715 39259733 PMC11389933

[48] Liao S Tian X Liu Z Liu X Chen O. Decision-making about fertility preservation after cancer diagnosis: a qualitative study of Patients’ experiences and perspectives. Cancer Manag Res. (2025) 17:2403–15. 10.2147/CMAR.S543626 41116783 PMC12535711

[49] Wang M Zhu L Xiong H Wang J Li Z Yang L Lack of knowledge, the main stumbling block of fertility preservation promotion in China. J Cancer Educ. (2020) 37(3):739–47. 10.1007/s13187-020-01875-2 32920747

[50] Monash IVF. (n.d.). *Egg freezing—fertility and egg preservation*. Available online at: https://monashivf.com/services/preserving-fertility/egg-freezing/ (Accessed January 20, 2026).

[51] Wang H. Single women’s access to egg freezing in mainland China: an ethicolegal analysis. J Med Ethics. (2024) 50(1):50. 10.1136/jme-2023-108915 37147115

[52] HKU-QMH CARE. (n.d.). *Charging: Public fertility preservation service fees*. Available online at: https://hkuivf.hku.hk/en/services/fertility-preservation/charging/ (Accessed January 20, 2026).

[53] India IVF Fertility. (2024). *Egg freezing cost in India: Everything you need to know*. Available online at: https://www.indiaivf.in/blog/egg-freezing-cost-in-india/ (Accessed January 20, 2026).

[54] Ono M Takai Y Harada M Horie A Dai Y Kikuchi E Out-of-pocket fertility preservation expenses: data from a Japanese nationwide multicenter survey. Int J Clin Oncol. (2024) 29(12):1959–66. 10.1007/s10147-024-02614-z 39231915 PMC11588863

[55] Jo I. Support for egg and Sperm Freezing for Expected Permanent Infertility… up to 2 Million won for Women. The Asia Business Daily (2025). Available online at: https://www.asiae.co.kr/en/article/2025042816442020121

[56] Busan Metropolitan City. Busan City Launches Support Program for Cryopreservation of Eggs and Sperm for Individuals Facing Predicted Permanent Infertility [Press Release]. City of Busan English AI-translated Press Releases (2025). Available online at: https://www.busan.go.kr/eng/ai-translated-press-releases/1679177 (Accessed January 20, 2026).

[57] Employees Provident Fund of Malaysia. (n.d.). *Fertility treatment*—Healthcare. KWSP-Gov.My. Available online at: https://www.kwsp.gov.my/en/member/healthcare/fertility (Accessed January 20, 2026).

[58] Factor PAA Novero VM Jr. Knowledge, attitudes, and practices of Filipino clinical practitioners regarding fertility preservation in cancer patients. Philipp J Obstet Gynecol. (2020) 44(3):12–21.

[59] Ministry of Health Singapore. (2025). Subsidies or Medisave withdrawals for egg freezing. Available online at: https://www.moh.gov.sg/newsroom/subsidies-or-medisave-withdrawals-for-egg-freezing (Accessed January 20, 2026).

[60] NUWA Fertility Center. (2025). 2024 *Egg freezing subsidy program*—Health education. NUWAcare.com. Available online at: https://www.nuwacare.com/en/health-education/2024-Egg-Freezing-Subsidy-Program (Accessed January 20, 2026).

[61] Mattawanon N Kummaraka U Oon-Arom A Manojai N Tangpricha V. Reproductive desires in transgender and gender diverse adults: a cross-sectional study in Thailand. Inte J Transgend Health. (2021) 23(3):362–74. 10.1080/26895269.2020.1864560 PMC9255026 35799958

[62] MEDLATEC. Tìm Hiểu chi Tiết chi phí trữ Đông Trứng và Những lưu ý khi Thực Hiện [Detailed Information About the Cost of egg Cryopreservation and Important Considerations]. MEDLATEC (2024). Available online at: https://medlatec.vn/tin-tuc/tim-hieu-chi-tiet-chi-phi-tru-dong-trung-va-nhung-luu-y-khi-thuc-hien

[63] Chin AHB. Singapore Needs to update regulation of frozen egg donation after permitting social egg freezing. J Assist Reprod Genet. (2022) 39(7):1497–500. 10.1007/s10815-022-02526-9 35653043 PMC9365903

[64] Rajvanshi A. Why China Won’t Allow Single Women to Freeze Their Eggs. TIME (2024). Available online at: https://time.com/7010985/china-egg-freezing-teresa-xu-ivf/

[65] Sun J. The social and legal implications of elective egg freezing in China. Peking Univ Law J. (2024) 12(1):41–60. 10.1080/20517483.2024.2400790

[66] Hong Kong Special Administrative Region Government. Legislative Council Q&A: Assisted Reproductive Services [Press Release]. Government of the Hong Kong Special Administrative Region (2023). Available online at: https://www.info.gov.hk/gia/general/202305/17/P2023051700447.htm

[67] Team Progenesis. Egg Freezing in India: Protocols, Costs, and Success Rates for Working Women. Progenesis IVF (2025). Available online at: https://progenesisivf.com/blog/egg-freezing-in-india/

[68] Otahara N. (2024). Tokyo to cover all eligible applicants for freezing eggs. Available online at: https://www.asahi.com/ajw/articles/15194646 (Accessed January 20, 2026).

[69] Choi J. Seoul Expands egg-freezing Subsidies, Doubling Beneficiaries to 650 Women. The Korea Herald (2024). Available online at: https://www.koreaherald.com/view.php?ud=20240214050618

[70] Muhsin SM Mohamad CAC Heng Boon Chin A. (2023). *Should Malaysian Muslim women undergo egg freezing overseas? Ova* (Opinion). Available online at: https://ova.galencentre.org/should-malaysian-muslim-women-undergo-egg-freezing-overseas-sayyed-mohamed-muhsin-dr-che-anuar-che-mohamad-dr-alexis-heng-boon-chin/ (Accessed January 20, 2026).

[71] Zora Health. Egg Freezing & IVF in the Philippines. Zora Health (n.d.). Available online at: https://zorahealth.co/locations/philippines/

[72] Heng Boon Chin A. Singapore can Justify Public egg Freezing Subsidy by Imposing Strict Conditions. BioNews, Progress Educational Trust (2024). Available online at: https://www.progress.org.uk/singapore-can-justify-public-egg-freezing-subsidy-by-imposing-strict-conditions/

[73] Bangkokfertilitycenter. Egg Freezing Cost in Thailand: Affordable Options for Fertility Preservation. Bangkok Fertility Centre (2024). Available online at: https://bangkokfertilitycenter.com/blog/egg-freezing-cost-in-thailand/

[74] Phan D. Preserving Fertility: Single Women Increasingly Turn to Freezing Eggs. VnExpress International (2024). Available online at: https://e.vnexpress.net/news/life/trend/preserving-fertility-single-women-increasingly-turn-to-freezing-eggs-4785898.html

[75] Yow SHQ. (2021). Why Does the Singaporean Government Care About Egg Freezing? Available online at: https://foreignpolicy.com/2021/10/19/singapore-egg-freezing-ban-law-population-social-control-demographics/ (Accessed January 20, 2026).

[76] Platts S Trigg B Bracewell-Milnes T Jones BP Saso S Parikh J Exploring women’s attitudes, knowledge, and intentions to use oocyte freezing for non-medical reasons: a systematic review. Acta Obstet Gynecol Scand. (2021) 100:383–93. 10.1111/aogs.14030 33078391

[77] Human Fertilisation and Embryology Authority. (2018). Egg freezing in fertility treatment. Available online at: https://www.hfea.gov.uk/treatments/fertility-preservation/egg-freezing/

[78] The World Bank. Population, Total (indicator SP.POP.TOTL). World Bank Open Data (n.d.). Available online at: https://data.worldbank.org/indicator/SP.POP.TOTL

[79] Ma Y Bai F Gao L Fan Y. The distribution and accessibility of assisted reproductive technology clinics in mainland China from 2006 to 2018: a population-based retrospective study. Hum Fertil. (2023) 26:573–81. 10.1080/14647273.2021.1969043 34412563

[80] Lazzari E Baffour B Chambers GM. Residential proximity to a fertility clinic is independently associated with likelihood of women having ART and IUI treatment. Hum Reprod. (2022) 37:2662–71. 10.1093/humrep/deac205 36112009 PMC9627258

[81] Khan HL Boothroyd C Chang TA Novero V Chan DYL Chen CH ASPIRE Guidelines for assisted reproductive technology (ART) laboratory practice in low and medium resource settings. Fertility Reprod. (2023) 5(3):115–33. 10.1142/S2661318223500184

[82] Amodini KN Chaudhuri S. Infertility management in India: issues and potential solutions. J Obstet Gynaecol India. (2023) 73(4):368–9. 10.1007/s13224-023-01814-3 37701090 PMC10492707

[83] Services Australia. MBS billing for Assisted Reproductive Technology Services. Australian Government (2025). Available online at: https://www.servicesaustralia.gov.au/mbs-billing-for-assisted-reproductive-technology-services?context=20

[84] National Healthcare Security Administration [国家医疗保障局]. (2025). *Doing in-vitro fertilisation (IVF) can now be reimbursed by medical insurance* [做试管婴儿也能医保报销了]. Available online at: https://www.nhsa.gov.cn/art/2025/2/26/art_14_15799.html (Accessed January 20, 2026).

[85] ART Unit, The Chinese University of Hong Kong & Prince of Wales Hospital. Appointment—public Service. IVFHK (n.d.). Available online at: https://www.ivfhk.com/en/public-service/

[86] Agency for Children and Families (Japan) [こども家庭庁]. (2023). *不妊治療保険適用リーフレット* [Infertility Treatment Insurance Coverage Leaflet]. Accessed from: h Available online at: ttps://www.cfa.go.jp/assets/contents/node/basic_page/field_ref_resources/bef0ee9a-c14d-4203-b02b-051adf80f495/cf3a6623/20230401_policies_boshihoken_funin_01.pdf (Accessed January 20, 2026).

[87] Choi SM. (2025). *2025년 국가 난임부부 시술비 지원사업 상세 설명* [Detailed explanation of the 2025 national infertility treatment support program]. Unknowing. Available online at: https://unknowing.tistory.com/entry/2025%EB%85%84-%EA%B5%AD%EA%B0%80-%EB%82%9C%EC%9E%84%EB%B6%80%EB%B6%80-%EC%8B%9C%EC%88%A0%EB%B9%84-%EC%A7%80%EC%9B%90%EC%82%AC%EC%97%85-%EC%83%81%EC%84%B8-%EC%84%A4%EB%AA%85 (Accessed January 20, 2026).

[88] Khoo GS. Perak Govt Introduces RM600,000 Subsidy for Childless Couples for IVF Treatment. The Star (2025). Available online at: https://www.thestar.com.my/news/nation/2025/02/22/perak-govt-introduces-rm600000-subsidy-for-childless-couples-for-ivf-treatment

[89] Ministry of Health, Singapore. Marriage and Parenthood Schemes. Singapore: Government of Singapore (2025). Available online at: https://www.moh.gov.sg/managing-expenses/schemes-and-subsidies/marriage-and-parenthood-schemes

[90] Chen MJ Kotsopoulos N Ming-Fang Yen A Lin KT Connolly MP. Estimating the public economic gains in Taiwan from *in vitro* fertilization (IVF) subsidy changes implemented in 2021. Hum Reprod. (2025) 40(2):328–34. 10.1093/humrep/deae271 39673722 PMC11788206

[91] Bangkok Post. (2024). B30 fertility treatment to tackle low birth rate. Available online at: https://www.bangkokpost.com/thailand/general/2764019/b30-fertility-treatment-to-tackle-low-birth-rate (Accessed January 20, 2026).

[92] The Economist Intelligence Unit. From Cubs to Ageing Tigers: Fertility in South-East Asia (EIU Report). The Economist Intelligence Unit (2019). Available online at: https://www.eiu.com/graphics/marketing/pdf/Fertility-in-South-East-Asia-EIU.pdf

[93] Kidd S Athias D Nastasi S Pop A. Inequality and Social Security in the Asia-Pacific region. Bangkok: United Nations Development Programme (2022).

[94] Assisted Reproduction Act. (2018). Available online at: https://law.moj.gov.tw/ENG/LawClass/LawAll.aspx?pcode=L0070024

[95] Health Promotion Administration. (2021). Expanded Subsidy for infertility treatment (IVF) takes effect on July 1. Available online at: https://www.hpa.gov.tw/EngPages/Detail.aspx?nodeid=1052&pid=14685 (Accessed January 20, 2026).

[96] Warne E Oxlad M Best T. Evaluating group psychological interventions for mental health in women with infertility undertaking fertility treatment: a systematic review and meta-analysis. Health Psychol Rev. (2023) 17:377–401. 10.1080/17437199.2022.2058582 35348050

[97] Shin H Lee J Kim S-J Jo M. Associations of symptoms of depression, social support, and quality of life among Korean women who experience infertility. J Obstet Gynecol Neonatal Nurs. (2021) 50:e1–e12. 10.1016/j.jogn.2021.06.007 34310903

[98] Xu Z Gahr M Xiang Y Kingdon D Rüsch N Wang G. The state of mental health care in China. Asian J Psychiatr. (2022) 69:102975. 10.1016/j.ajp.2021.102975 34998231

[99] Murray S Awartani KA Péloquin S. International challenges in patient-centred care in fertility clinics offering assisted reproductive technology: providers’ gaps and attitudes towards addressing the patients’ psychological needs. J Eur CME. (2015) 4:27578. 10.3402/jecme.v4.27578

[100] International Infertility Counselling Organisation. List of national infertility counseling organizations. Available online at: https://www.iico-infertilitycounseling.org/1216-2/ (Accessed January 20, 2026).

[101] Daar J Benward J Collins L Davis J Davis O Francis L Use of preimplantation genetic testing for monogenic defects (PGT-M) for adult-onset conditions: an ethics committee opinion. Fertil Steril. (2018) 109:989–92. 10.1016/j.fertnstert.2018.04.003 29935659

[102] Jin H Zheng H Salbato AN Snyder R Liu L. Development of a next generation sequencing method (PGT-SR plus) to determine carrier status of balanced translocation patient embryos. Fertil Steril. (2019) 112:e242–3. 10.1016/j.fertnstert.2019.07.1378

[103] Lensen S Hammarberg K Polyakov A Wilkinson J Whyte S Peate M How common is add-on use and how do patients decide whether to use them? A national survey of IVF patients. Hum Reprod. (2021) 36:1854–61. 10.1093/humrep/deab098 33942073

[104] Wilkinson J Malpas P Hammarberg K Mahoney Tsigdinos P Lensen S Jackson E Do à la carte menus serve infertility patients? The ethics and regulation of *in vitro* fertility add-ons. Fertil Steril. (2019) 112:973–7. 10.1016/j.fertnstert.2019.09.028 31703942

[105] Hughes T Bracewell-Milnes T Saso S Jones BP Almeida PA Maclaren K A review on the motivations, decision-making factors, attitudes and experiences of couples using pre-implantation genetic testing for inherited conditions. Hum Reprod Update. (2021) 27:944–66. 10.1093/humupd/dmab013 33969393

[106] Aguilar AS Novero VJ Ong-Jao ET Utulo M Cortes-Gaspar TA Enriquez-Gamboa M Agreement with “the ethical guidelines on the provision and practice of advanced reproductive technology and intrauterine insemination 2023” by the Philippine society of reproductive medicine using online delphi technique. Glob Reprod Health. (2024) 9. 10.1097/GRH.0000000000000091

[107] Newman JE Paul RC Chambers GM. Assisted Reproductive Technology in Australia and New Zealand 2021 (Annual Report). Sydney: National Perinatal Epidemiology and Statistics Unit, UNSW Sydney (2023). Available online at: https://www.unsw.edu.au/content/dam/pdfs/medicine-health/npesu/research-reports/2023-12-npesu/2024-01-Assisted-Reproductive-Technology-in-Australia-and-New-Zealand-2021.pdf

[108] Australian Government Department of Health and Aged Care. Pre-implantation Genetic Testing (PGT) Factsheet. Medicare Benefits Schedule Online (2021). Available online at: https://www.mbsonline.gov.au/internet/mbsonline/publishing.nsf/Content/Factsheet-Pre-imp-Gentest

[109] Yan L Cao Y Chen ZJ Du J Wang S Huang H Chinese Experts’ consensus guideline on preimplantation genetic testing of monogenic disorders. Hum Reprod. (2023) 38(2):ii3–ii13. 10.1093/humrep/dead112 37982416

[110] He X Wang X Shen J Zhang Q Liu Y Li B. Cost-effectiveness of preimplantation genetic testing for aneuploidy for women with subfertility in China: an economic evaluation using evidence from the CESE-PGS trial. BMC Pregnancy Childbirth. (2023) 23:254. 10.1186/s12884-023-05563-z 37060068 PMC10103395

[111] HKU-QMH CARE. Charging—preimplantation Genetic Testing (PGT). The Centre of Assisted Reproduction and Embryology, The University of Hong Kong-Queen Mary Hospital (n.d.). Available online at: https://hkuivf.hku.hk/en/services/preimplantation-genetic-testing/charging/

[112] Indira IVF. (n.d.). *Preimplantation genetic testing/diagnosis* (PGT-A) cost | Indira IVF. Available online at: https://www.indiraivf.com/advanced-infertility-treatment/preimplantation-genetic-testing-diagnosis-pgta-cost/ (Accessed January 20, 2026).

[113] Amelia F. Mengenal Preimplantation Genetic Testing (PGT) [Understanding Preimplantation Genetic Testing (PGT)]. Bocah Indonesia (2023). Available online at: https://bocahindonesia.com/preimplantation-genetic-testing/

[114] Tamura C. Regulation of the preimplantation genetic testing in Japan: challenges for the clinical application. Reprod Biomed Online. (2019) 38(1):E57–58. 10.1016/J.RBMO.2019.03.091

[115] Harada S Yamada M Shirasawa H Jwa SC Kuroda K Harada M Fact-finding survey on assisted reproductive technology in Japan. J Obstet Gynaecol Res. (2023) 49(11):2593–601. 10.1111/jog.15780 37635650

[116] Aizawa Y Watanabe A Kato K. Institutional and social issues surrounding genetic counselors in Japan: current challenges and implications for the global community. Front Genet. (2021) 12:646177. 10.3389/fgene.2021.646177 33927749 PMC8076856

[117] Ministry of Health and Welfare (Republic of Korea). (2009). *배아·태아 대상 유전자검사 허용 유전질환 139종으로 확대* [Expansion of the 139 genetic diseases eligible for embryo and fetus genetic testing]. Available online at: https://www.mohw.go.kr/board.es?mid=a10503000000&bid=0027&act=view&list_no=218124 (Accessed January 20, 2026).

[118] Lee SH. 400,000 won per Injection? Government Infertility Support not Felt. Asiae—The Asia Business Daily (2024). Available online at: https://cm.asiae.co.kr/en/article/2024021318322186900

[119] Olesen AP Mohd Nor SN Amin L Che Ngah A. Public perceptions of ethical, legal and social implications of pre-implantation genetic diagnosis (PGD) in Malaysia. Sci Eng Ethics. (2017) 23(6):1563–80. 10.1007/s11948-016-9857-z 27995446

[120] Abad PJ Tumulak MJ Guerbo R de Castro-Hamoy L Bautista NG Nuique R Landscape of genetic counseling in the Philippines. J Genet Couns. (2024) 33(5):934–42. 10.1002/jgc4.1804 37877196

[121] Health Promotion Administration, Ministry of Health and Welfare, Taiwan. (n.d.). *體外受精(俗稱試管嬰兒)人工生殖技術補助方案民眾申辦作業說明* [Assistance program application instructions for *in-vitro* fertilization (commonly known as IVF)]. Available online at: https://www.hpa.gov.tw/Pages/Detail.aspx?nodeid=500&pid=14294 (Accessed February 10, 2026).

[122] Bumrungrad International Hospital. (2026). *Preimplantation genetic testing (PGT)*. Available online at: https://www.bumrungrad.com/en/treatments/preimplantation-genetic-testing-pgt (Accessed January 20, 2026).

[123] Trường Thịnh. (2024). *Sàng lọc phôi tiền làm tổ giúp bố mẹ mắc bệnh di truyền sinh con khỏe mạnh* [Preimplantation embryo screening helps parents with genetic disorders have healthy children]. *Dân trí*. Available online at: https://dantri.com.vn/suc-khoe/sang-loc-phoi-tien-lam-to-giup-bo-me-mac-benh-di-truyen-sinh-con-khoe-manh-20240507163824327.htm (Accessed January 20, 2026).

[124] Olesen AP Nor SNM Amin L. Attitudes toward Pre-implantation genetic diagnosis (PGD) for genetic disorders among potential users in Malaysia. Sci Eng Ethics. (2016) 22:133–46. 10.1007/s11948-015-9639-z 25724710

[125] Armstrong S Atkinson M MacKenzie J Pacey A Farquhar C. Add-ons in the laboratory: hopeful, but not always helpful. Fertil Steril. (2019) 112:994–9. 10.1016/j.fertnstert.2019.10.031 31843099

[126] Zegers-Hochschild F Adamson GD de Mouzon J Ishihara O Mansour R Nygren K International committee for monitoring assisted reproductive technology (ICMART) and the world health organization (WHO) revised glossary of ART terminology, 2009. Fertil Steril. (2009) 92(5):1520–4. 10.1016/j.fertnstert.2009.09.009 19828144

[127] Australian Government, Centre for Population. (2022). Impacts of policies on fertility rates. Available online at: https://population.gov.au/sites/population.gov.au/files/2022-03/ANU_Impacts-of-Policies-on-Fertility-Rates-Overview.pdf (Accessed January 20, 2026).

[128] Ipsos Australia & Organon. Organon Women’s Fertility Study Report (2023). (Accessed January 20, 2026).

[129] Igaabap P Batiari NMP Aswari NWC. The effectiveness of health education and media using local language in improving reproductive health knowledge among adolescents. In: Proceedings of the International Conference on Public Health, Vol. 8, Issue 2. TIIKM Publishing (2023). p. 47–58. 10.17501/24246735.2023.8206

[130] Takae S Lee JR Mahajan N Wiweko B Sukcharoen N Novero V Fertility preservation for child and adolescent cancer patients in Asian countries. Front Endocrinol. (2019) 10:655. 10.3389/fendo.2019.00655 PMC6804405 31681163

[131] Harzif AK Santawi VPA Maidarti M Wiweko B. Investigation of each society for fertility preservation in Asia. Front Endocrinol. (2019) 10:151. 10.3389/fendo.2019.00151 PMC6426758 30923515

[132] Ayuandari S Khasanah N Riyanti IW Dewanto A Enisar Sangun DI Wiweko B. Current awareness and attitude toward fertility preservation in Indonesia: a nationwide survey among health-care providers. J Hum Reprod Sci. (2021) 14(1):81–6. 10.4103/jhrs.jhrs_239_20 34083997 PMC8057139

[133] Tanzola M. (2025). *ASCO updates guideline for fertility preservation in people with cancer*. *The ASCO Post*. Available online at: https://ascopost.com/issues/april-25-2025/asco-updates-guideline-for-fertility-preservation-in-people-with-cancer/ (Accessed February 18, 2026).

[134] Yeung SY Ng EYL Lao TTH Li TC Chung JPW. Fertility preservation in Hong Kong Chinese society: awareness, knowledge and acceptance. BMC Women’s Health. (2020) 20(1):86. 10.1186/s12905-020-00953-3 32349724 PMC7189503

[135] Johnston M Fuscaldo G Gwini SM Catt S Richings NM. Financing future fertility: Women’s views on funding egg freezing. Reprod Biomed Soc Online. (2022) 14:32–41. 10.1016/j.rbms.2021.07.001 34693043 PMC8517713

[136] Pawa R Udomsrisumran L Kiatpongsan S. Fertility Physicians’ opinions and attitudes on access to assisted reproductive technology: an Asia-Pacific perspective. Fertil Reprod. (2020) 02:61–9. 10.1142/S2661318220500097

[137] Jung-joo L. (2024). Seoul to help more women to freeze eggs. Available online at: https://www.koreaherald.com/article/3325931 (Accessed January 20, 2026).

[138] Yokoyama M Lee MJ. (2024). Tokyo’s Fertility Program Is Overwhelmed by How Many Women Want to Freeze Their Eggs. Available online at: https://time.com/6564312/japan-tokyo-egg-freezing-women-demand-fertility-subsidy/ (Accessed January 20, 2026).

[139] Rodrigues-Martins D Vale-Fernandes E Leal C Barreiro M. Influence of Womeńs residence region on assisted reproduction treatments—experience of a tertiary center in northern Portugal. JBRA Assist Reprod. (2022) 26(1):73–7. 10.5935/1518-0557.20210059 34609110 PMC8769178

[140] Lai S. (2024). Expanded IVF subsidy program marks 3rd anniversary with over 20,000 newborns. Available online at: https://focustaiwan.tw/society/202408190015 (Accessed January 20, 2026).

[141] Chambers GM Hoang VP Zhu R Illingworth PJ. A reduction in public funding for fertility treatment—an econometric analysis of access to treatment and savings to government. BMC Health Serv Res. (2012) 12:142. 10.1186/1472-6963-12-142 22682009 PMC3464128

[142] Connolly MP Hoorens S Chambers GM. The costs and consequences of assisted reproductive technology: an economic perspective. Hum Reprod Update. (2010) 16:603–13. 10.1093/humupd/dmq013 20530804

[143] Keller E Botha W Chambers GM. Does *in vitro* fertilization (IVF) treatment provide good value for money? A cost-benefit analysis. Front Glo Womens Health. (2023) 4:971553. 10.3389/fgwh.2023.971553 PMC10014591 36937042

[144] Iordăchescu DA Golu FT Paica CI Gorbănescu A Panaitescu AM Gică C The relationship between the infertility specialist and the patient during the COVID-19 pandemic. Healthcare. (2021) 9:1649. 10.3390/healthcare9121649 34946375 PMC8702128

[145] Department of Health, Victoria. (2024). Public fertility care services. Available online at: https://www.health.vic.gov.au/public-health/public-fertility-care-services (Accessed January 20, 2026).

[146] IVF Australia. Counselling support and advice when you need it most. Available online at: https://www.ivf.com.au/specialists/meet-our-counsellors (Accessed January 20, 2026).

[147] Health and Medical Affairs. Bioethics and Safety Act. Ministry of Health and Welfare (2017).

